# Uncoupling Type I Interferon Benefits From Inflammatory Toxicity: Transformer‐Prioritized Precision Agonists for Potent and Safer Cancer Immunotherapy

**DOI:** 10.1002/advs.77269

**Published:** 2026-08-24

**Authors:** Xuefei Guo, Yang Zhao, Xianle Rong, Xingyu Chen, Xiao Wang, Tianyi Liu, Yunfei Xie, Yushu Zou, Pingsen Zhao, Qiang Liu, Fuping You

**Affiliations:** ^1^ Department of Laboratory Medicine Yuebei People's Hospital Affiliated to Shantou University Medical College Shaoguan China; ^2^ Institute of Systems Biomedicine School of Basic Medical Sciences NHC Key Laboratory of Medical Immunology Peking University Health Science Center Beijing China; ^3^ MOE Key Laboratory of Bioinformatics and Bioinformatics Division BNRIST Department of Automation Tsinghua University Beijing China; ^4^ Center of Basic Molecular Science (CBMS) Department of Chemistry Tsinghua University Beijing China

**Keywords:** inflammatory response, innate immunity response, NF‐Κb, pancreatic ductal adenocarcinoma (PDAC), phosphines‐nitrogen‐phosphines (PNP)‐pincer ligands, STAT1, TLR4, Transformer

## Abstract

Paclitaxel (PTX) chemotherapy is constrained by an “immunomodulatory paradox,” where antitumor Type I Interferon (IFN‐I) activation is coupled with detrimental pro‐inflammatory cascades. To address this challenge, we developed Deep Learning for Innate Immunity Modulatory Potential (DLINP), a Transformer‐based framework designed to identify precision immunomodulators that uncouple IFN‐I induction from deleterious inflammatory signaling. Screening 123 million entities identified Co68—an organometallic PNP‐pincer complex—as a dual‐functional agent with superior potency to conventional taxanes. In pancreatic ductal adenocarcinoma (PDAC) models, Co68 elicited robust antitumor responses that exceeded those of the gold‐standard STING agonist DMXAA. Single‐cell and spatial transcriptomics revealed that Co68 selectively re‐engineered the myeloid compartment, reprogramming tumor‐associated macrophages toward an interferon‐stimulated gene (ISG)‐high phenotype while quenching the pro‐inflammatory IL1β–PGE2 feedback loop. This reconfiguration converted “cold” tumor microenvironments into “hot” landscapes, enhancing NK and CD8^+^ T cell recruitment and synergy with anti–PD‐1 therapy. Mechanistically, Co68 engages the TLR4–MD2 complex via a non‐canonical binding mode, bifurcating innate signaling: triggering the TLR4–TRIF–IFN‐I axis while attenuating NF‐κB‐driven inflammation through an early, IFNAR‐independent TLR4–SYK–STAT1 pathway. Collectively, Co68 represents a taxane‐inspired precision therapeutic that uncouples beneficial antiviral‐like immunity from pathogenic inflammation, offering a transformative strategy for refractory solid tumors.

## Graphical abstract

A Transformer‐based AI framework, DLINP, screens millions of compounds to identify Co68, a cobalt‐pincer organometallic complex that biases TLR4‐MD2 signaling toward antitumor interferon activation while suppressing inflammatory toxicity through an early TLR4‐SYK‐STAT1 axis. In pancreatic cancer models, Co68 reprograms tumor‐associated macrophages, disrupts the IL‐1β‐PGE2 circuit, and synergizes with anti‐PD‐1 therapy, thereby establishing a precision strategy for safer immunotherapy.

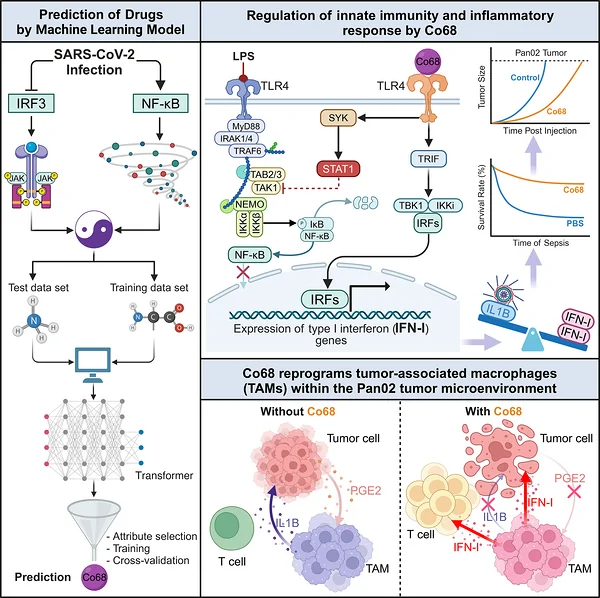

## 1 Introduction

Pancreatic ductal adenocarcinoma (PDAC) remains one of the deadliest malignancies, with a five‐year survival rate of approximately 10% and a projected rise to the second leading cause of cancer‐related mortality by 2030 [1, 2, 3]. Despite advances in surgery, chemotherapy, and targeted therapies, clinical outcomes remain poor, largely owing to the profoundly desmoplastic and immunosuppressive tumor microenvironment (TME) [4]. Within this ecosystem, tumor‐associated macrophages (TAMs) orchestrate chronic inflammation by producing interleukin‐1β (IL‐1β), which forms a reciprocal positive‐feedback circuit with tumor‐derived prostaglandin E2 (PGE2) [5, 6, 7]. This IL‐1β–PGE2 inflammatory loop promotes tumor progression, immune evasion, and therapeutic resistance, highlighting the need for therapeutic strategies that simultaneously remodel myeloid inflammation and restore antitumor immunity [8, 9, 10, 11].

Paclitaxel (PTX), a cornerstone therapy for PDAC, has recently been recognized as an immunomodulatory ligand of the TLR4–MD2 complex in addition to its canonical microtubule‐stabilizing activity [12, 13, 14]. TLR4 activation by PTX induces type I interferon (IFN‐I), thereby promoting innate and adaptive antitumor immunity [13]. However, the same signaling event simultaneously activates the MyD88–NF‐κB pathway, resulting in excessive IL‐1β production and persistent inflammatory toxicity [15, 16, 17]. Consequently, the beneficial effects of IFN‐I are intrinsically coupled to pathogenic inflammation, limiting therapeutic durability [18, 19, 20]. This immunomodulatory paradox represents a fundamental challenge in the development of innate immune agonists: how to selectively activate TLR4‐driven IFN‐I signaling while minimizing NF‐κB‐mediated inflammatory responses.

Machine learning (ML) is increasingly reshaping drug discovery by linking large chemical spaces with high‐dimensional biological readouts to prioritize candidates with desired therapeutic profiles [21, 22, 23]. Although conventional ML approaches have improved molecular property prediction and target identification, they often rely on predefined features and may have limited capacity to capture long‐range structural dependencies or context‐dependent signaling relationships [24, 25, 26, 27]. Transformer‐based architectures address these limitations through self‐attention mechanisms that model global molecular features and complex structure‐activity relationships directly from sequence‐based representations [28, 29, 30, 31, 32]. This capability makes Transformers well suited for discovering precision immunomodulators whose activity depends on coordinated regulation of multiple immune pathways [26].

Here, we developed Deep Learning for Innate Immunity Modulatory Potential (DLINP), a Transformer‐based framework designed to identify small molecules capable of functionally uncoupling IFN‐I activation from inflammatory amplification. Using DLINP, we identified Co68, a PNP‐pincer organometallic complex with potent dual immunomodulatory activity. Co68 reprogrammed tumor‐associated myeloid cells toward an interferon‐stimulated phenotype while suppressing the IL‐1β–PGE2 inflammatory circuit, thereby converting immunologically “cold” tumors into inflamed, immune‐responsive tumors. Mechanistically, Co68 engaged the TLR4–MD2 complex through a previously unrecognized binding mode to activate the TLR4–TRIF–IFN‐I pathway, while simultaneously suppressing inflammation through an early TLR4–SYK–STAT1 signaling axis independent of IFNAR signaling. Together, these findings establish Co68 as a precision innate immune agonist that selectively uncouples beneficial IFN‐I signaling from pathogenic inflammation and provide a conceptual framework for the rational design of safer cancer immunotherapies.

## 2 Results

### 2.1 Spatial Landscape and Clinical Significance of the DIMI Framework

**FIGURE 1:**
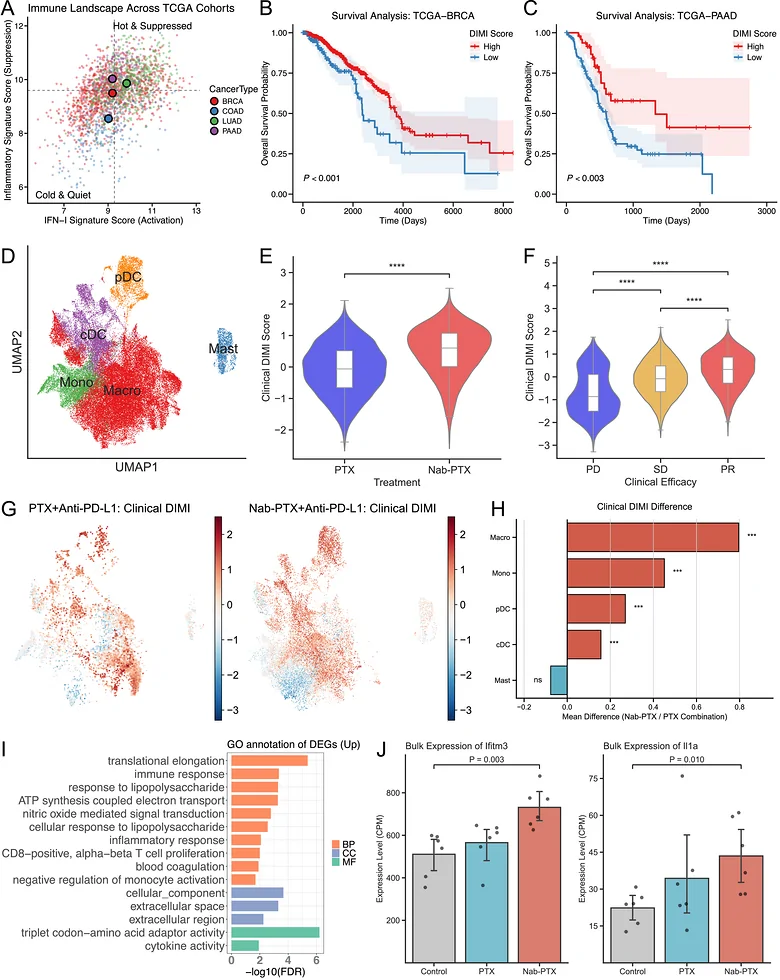
The dimi index as a decisive factor for tumor immunological stratification and therapeutic responsiveness. (A) Quadratic stratification of tumor immunological landscapes. Pan‐cancer cohorts from TCGA were categorized into four quadrants—“Hot and Suppressed”, “Cold”, “IFN‐I Low/Inflammation Low”, and “Optimal TME”—based on the relative expression of Type I Interferon (IFN‐I) and inflammatory signature scores. (B, C) Prognostic value of the DIMI index. Kaplan‐Meier survival curves for patients in the BRCA (B) and PAAD (C) cohorts, stratified by median DIMI scores. P‐values were determined by the log‐rank test. (D) Umap of the myeloid cells from (GSE266919). Breast cancer patients received treatment with Paclitaxel (PTX) or albumin‐bound paclitaxel (Nab‐PTX) in combination with anti‐PD‐L1 therapy. (E) Therapeutic modulation of DIMI scores. Comparison of the DIMI index distribution between the PTX and Nab‐PTX treatment arms. (F) Correlation between DIMI scores and clinical response. DIMI indices are compared across patients with Partial Response (PR), Stable Disease (SD), and Progressive Disease (PD). (G) Cell‐type‐specific DIMI profiling. UMAP visualization and DIMI score distribution across major immune cell compartments in the tumor microenvironment (TME) following Nab‐PTX + anti‐PD‐L1 therapy. (H) Myeloid lineage response to Nab‐PTX. Comparison of DIMI scores within specific myeloid sub‐clusters, including Macrophages, Monocytes, pDCs, and cDCs, between PTX and Nab‐PTX groups. (I) Functional enrichment analysis of Nab‐PTX‐induced transcriptomes. Gene Ontology (GO) enrichment analysis of significantly upregulated genes in the Nab‐PTX group compared to the control, highlighting pathways related to LPS response and inflammation. (J) Dichotomous gene expression profiling. Bar plot showing the concurrent upregulation of canonical IFN‐stimulated genes (ISGs) and pro‐inflammatory cytokines in the Nab‐PTX group. Data are presented as mean ± SEM. Statistical significance was evaluated by a two‐tailed Student's *t*‐test or one‐way ANOVA. ^*^ *p* < 0.05, ^**^ *p* < 0.01, ^***^ *p* < 0.001.

To systematically characterize the immunological landscapes across diverse malignancies, we stratified TCGA cohorts into four distinct quadrants based on their Type I Interferon (IFN‐I) and inflammatory signature scores (Figure 1A). We identified that LUAD resides in the “Hot and Suppressed” quadrant, characterized by elevated IFN‐I levels coupled with intense inflammatory activity, suggesting that mitigating inflammation‐mediated immunosuppression may synergize with anti‐PD‐1/PD‐L1 blockade. Conversely, PAAD presents as a classic “Cold” tumor with deficient IFN‐I signaling and a robust pro‐inflammatory stroma, necessitating a dual strategy of IFN‐I induction and inflammatory modulation. In BRCA and COAD, which exhibit synchronized low‐inflammatory and low‐IFN‐I profiles, therapeutic efficacy may hinge on bolstering innate IFN‐I signaling to reinvigorate CD8^+^ T‐ and NK cell‐mediated cytotoxicity.

Guided by these observations, we proposed the Differential Interferon‐to‐Inflammatory (DIMI) index (defined as the IFN‐I signature score minus the inflammatory signature score) as a refined metric for molecular stratification. Survival analysis revealed that an elevated DIMI index significantly correlates with prolonged overall survival in BRCA, PAAD, and LUAD cohorts (Figure 1B,C and Figure S1A), establishing DIMI as a robust prognostic indicator across multiple solid tumors. The DIMI Index Predicts Clinical Responsiveness to Nano‐chemoimmunotherapy. To evaluate the clinical utility of the DIMI framework, we performed a deep‐dive analysis of a single‐cell RNA sequencing dataset (GSE266919) featuring breast cancer patients treated with either Paclitaxel (PTX) or its albumin‐bound nanoparticle formulation, Nab‐paclitaxel (Nab‐PTX), in combination with anti‐PD‐L1 (Figure 1D and Figure S1B,C). In line with the superior clinical efficacy observed in the Nab‐PTX arm, Nab‐PTX‐treated patients exhibited significantly higher DIMI scores than those in the PTX group (Figure 1E). Notably, the DIMI index was markedly elevated in responders vs. non‐responders (Figure S1D) and, specifically, in the Partial Response (PR) group compared with the Stable Disease (SD) or Progressive Disease (PD) cohorts (Figure 1F). Correspondingly, differentially expressed genes (DEGs) in the PR group were predominantly enriched in pathways associated with IFN‐α/β signaling and antiviral innate immunity (Figure S1E).

Myeloid‐specific DIMI Dynamics and the Limitations of Current Taxanes. At the cellular level, the Nab‐PTX/anti‐PD‐L1 combination uniquely modulated the DIMI dynamics within the myeloid compartment (Figure 1G). Specifically, Nab‐PTX significantly enhanced the DIMI indices of Macrophages, Monocytes, pDCs, and cDCs (Figure 1H). While Nab‐PTX effectively upregulated canonical IFN‐stimulated genes (ISGs) such as *HLA‐B, IFITM3, ISG15*, and *MX1* (Figure S1F), it also triggered a robust pro‐inflammatory program, as evidenced by elevated expression of *IL1B, IL6*, and *TNF* (Figure S1G). This concurrent activation was further validated by bulk RNA‐seq analysis, which showed that Nab‐PTX treatment induced gene sets significantly enriched for immune response to lipopolysaccharide and inflammatory response (Figure 1I and Figure S1H). Consistent with the single‐cell findings, Nab‐PTX treatment, while successfully augmenting IFN‐I potency (e.g., *IFITM3, MX1*, and *ISG15*), inevitably activated a spectrum of pro‐inflammatory cytokines, including *IL1A, IL1B*, and *TNF* (Figure 1J and Figure S1I). These results suggest that although Nab‐PTX, as a nanomedicine‐optimized taxane, offers superior immunostimulatory capacity, it still elicits a substantial inflammatory burden likely through the TLR4‐NF‐κB axis. This underscores an urgent need in advanced drug delivery: the development of next‐generation taxane formulations that precisely uncouple IFN‐I potentiation from detrimental inflammatory signaling to maximize the therapeutic index.

### 2.2 De Novo Identification of Dual‐Functional Immunomodulators via a Transformer‐Based Deep Learning Framework

**FIGURE 2:**
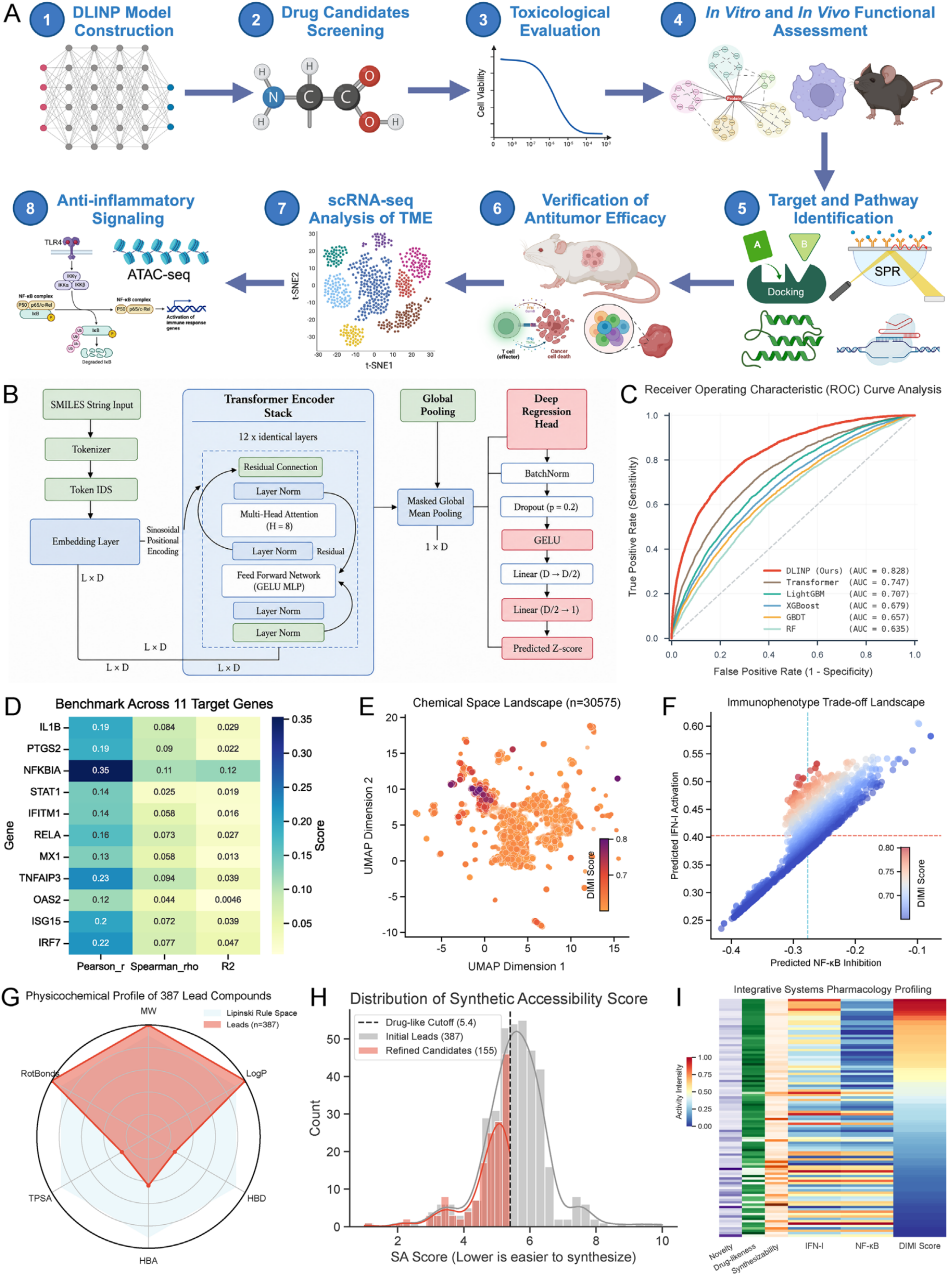
De novo identification of the lead immunomodulators via the DLINP framework. (A) Schematic workflow of the DLINP‐based discovery platform. The pipeline encompasses deep learning‐guided virtual screening of the PubChem database, hit prioritization, and multi‐layered experimental validation. (B) Architecture of the DLINP model. The framework utilizes a Transformer Encoder stack with Multi‐Head Attention (MHA) and Masked Global Mean Pooling to decode SMILES strings and predict gene expression Z‐scores. (C) Performance evaluation of the DLINP model. ROC curves and AUC values demonstrate the superior predictive accuracy of DLINP compared to conventional machine learning algorithms and vanilla Transformer models. (D) Weighted importance of target genes in the scoring framework. Pearson correlation coefficients highlight the contribution of individual genes, such as *Nfkbia*, to the prioritization process. (E) Large‐scale virtual screening results. Distribution of predicted DIMI scores across 123 million chemical entities from PubChem, identifying a sub‐cohort of molecules with high immunomodulatory potential. (F) Prioritization of high‐DIMI candidates. Scatter plot illustrating 387 compounds with DIMI scores > 0.7, predicted to simultaneously induce IFN‐I and suppress inflammation. (G) Pharmacological characterization and drug‐likeness. Distribution of prioritized candidates within the Lipinski‐defined chemical space, assessed by molecular weight and octanol‐water partition coefficient (logP). (H) Synthetic accessibility and multi‐parameter filtering. Convergence of predicted DIMI scores and SA scores (SA < 5.4) for the identification of 155 top‐tier candidates. (I) Summary of lead candidate properties. The heatmap depicts the integrated DIMI scores and drug‐likeness profiles of the 155 identified compounds.

These clinical and molecular observations underscored the urgent necessity to identify next‐generation compounds capable of bifurcating immune responses—specifically, those that potently induce IFN‐I production while simultaneously quenching detrimental inflammatory signaling. The systematic workflow for this identification process is delineated in Figure 2A. To navigate the vast chemical landscape, we engineered the Deep Learning for Innate Immunity Modulatory Potential (DLINP) model. This Transformer‐based architecture was deployed to perform a high‐throughput virtual screen of the PubChem database, which encompasses over 123 million unique chemical entities. The computational campaign prioritized a cohort of candidates predicted to achieve the desired dual‐modulatory phenotype. These top‐tier molecules underwent rigorous in vitro and in vivo validation to verify their capacity to antagonize NF‐κB‐mediated inflammation while amplifying IRF3‐dependent antiviral immunity. Among the evaluated candidates, a novel compound designated as “Co68” emerged as the lead agent, demonstrating superior efficacy and a favorable safety profile. Subsequent mechanistic investigations elucidated the molecular targets through which Co68 orchestrates IFN‐I activation. The therapeutic potential of Co68 was further scrutinized in a Pan02 pancreatic ductal adenocarcinoma mouse model utilizing single‐cell RNA sequencing (scRNA‐seq), which confirmed its robust pan‐anti‐inflammatory activity and established it as a promising candidate for immuno‐oncology applications.

To precisely extract immunomodulatory signatures from high‐dimensional chemical spaces, the DLINP framework was constructed upon a sophisticated Transformer Encoder architecture. This model is specifically designed to decode the non‐local inter‐atomic dependencies within SMILES strings and project them onto biological phenotypic manifolds. As illustrated in the workflow in Figure 2B, input SMILES sequences are tokenized and integrated with Sinusoidal Positional Encodings to encapsulate the essential topological and spatial arrangements of the molecular graphs. The architecture features a Transformer Encoder Stack consisting of 12 identical layers, each employing an 8‐head Multi‐Head Attention (MHA) mechanism to capture pharmacophoric features across multiple latent subspaces. To standardize the variable‐length molecular representations, we implemented a Masked Global Mean Pooling layer, which condenses high‐dimensional sequence data into a fixed‐length global feature vector. Finally, a Deep Regression Head, enhanced with Batch Normalization and Dropout layers, ensures the robust prediction of gene expression Z‐scores. Our benchmarking analysis indicates that this architecture provides exceptional feature extraction and generalization capabilities, particularly for complex, long‐chain organic molecules that often elude traditional descriptors. The predictive prowess of DLINP was rigorously quantified using confusion matrix analysis and Receiver Operating Characteristic (ROC) curves. As demonstrated in Figure 2C, the DLINP model achieved an Area Under the Curve (AUC) of 0.828, significantly surpassing conventional machine learning estimators—including LightGBM, XGBoost, GBDT, and Random Forest—as well as vanilla Transformer‐based screening protocols. These metrics underscore the superior reliability of DLINP in deciphering complex immune‐modulatory SAR (structure‐activity relationships). Following model optimization, eleven target genes were integrated into a weighted scoring matrix to evaluate the immunomodulatory potential of candidate molecules (Figure 2D). Within this framework, NFKBIA was identified as a pivotal determinant, exhibiting a Pearson correlation coefficient of 0.35, thereby exerting a dominant influence on the prioritization of molecules with anti‐inflammatory potential.

Leveraging the validated DLINP model, we executed a large‐scale screen of the PubChem library. As shown in Figure 2E, 30 575 molecules were identified with a DIMI score exceeding 0.6. Applying a more stringent threshold (DIMI > 0.7) narrowed the selection to 387 high‐priority compounds (Figure 2F), which were predicted to simultaneously activate IFN‐I pathways and suppress pro‐inflammatory cascades. To assess their translational potential, we performed a systematic pharmacological characterization. The majority of these 387 candidates occupied a chemical space consistent with Lipinski's Rule of Five (Figure 2G), reflecting favorable physicochemical attributes. Compared to the initial screening background, these prioritized molecules exhibited significantly optimized molecular weights, octanol‐water partition coefficients (logP), and hydrogen‐bond donor counts (Figure S2A), indicating enhanced drug‐likeness. Furthermore, synthetic accessibility (SA) filters identified 155 compounds with low complexity (SA score < 5.4). The convergence of predicted DIMI scores and drug‐like parameters for these 155 candidates is summarized in Figure 2I. Collectively, this multi‐stage hierarchical filtering framework enables the discovery of next‐generation, taxane‐inspired immunomodulators characterized by a potent dual‐functional profile: the robust induction of IFN‐I and the effective resolution of inflammation.

### 2.3 Co68 Induces Type I Interferon Signaling and Suppresses Inflammatory Responses

As illustrated in Figure S2B, several DLINP‐identified candidates effectively suppressed lipopolysaccharide (LPS)‐induced inflammatory cascades in RAW 264.7 macrophages. Among them, Co68 exhibited a superior dual‐modulatory profile, robustly activating *Ifnb1* expression while concurrently maintaining potent anti‐inflammatory activity (Figure S2C). The chemical architecture of Co68 is delineated in Figure S2D. Its synthesis involved coordinating a Phosphines–Nitrogen–Phosphines (PNP) pincer ligand—a scaffold common in homogeneous catalysis—with cobalt chloride in anhydrous tetrahydrofuran (THF) under an inert atmosphere. We next evaluated the capacity of Co68 to modulate *Ifnb1* across diverse macrophage lineages. Co68 treatment elicited robust upregulation of *Ifnb1* transcripts, exhibiting cell‐type‐specific dose dependencies. Maximal induction in both RAW 264.7 cells and primary bone marrow‐derived macrophages (BMDMs) occurred at 200 µm (Figure S2E). Temporal kinetic analyses revealed that *Ifnb1* expression peaked at 3 h post‐treatment in RAW 264.7 cells, with a slightly delayed zenith at 5 h in BMDMs (Figure S2F). Cytotoxicity profiling indicated that Co68 was well‐tolerated at therapeutic doses, with significant mortality observed only at supra‐pharmacological concentrations (500 µm) (Figure S2G). To assess systemic immunomodulatory potential in vivo, mice were administered 10 µm of Co68. After 24 h, significant induction of *Ifnb1* and the interferon‐stimulated gene *Ifit3* was detected in the spleen, lung, liver, and peripheral blood. Notably, no aberrant transcriptional changes occurred in the heart or kidney, suggesting favorable biosafety and biodistribution (Figure S2H). Based on this optimized therapeutic window, subsequent mechanistic studies were standardized at 100 µm Co68 for 3 h. Collectively, these findings demonstrate the efficacy and safety of Co68 in orchestrating innate immune responses, affirming the precision of the DLINP framework for de novo drug discovery.

**FIGURE 3:**
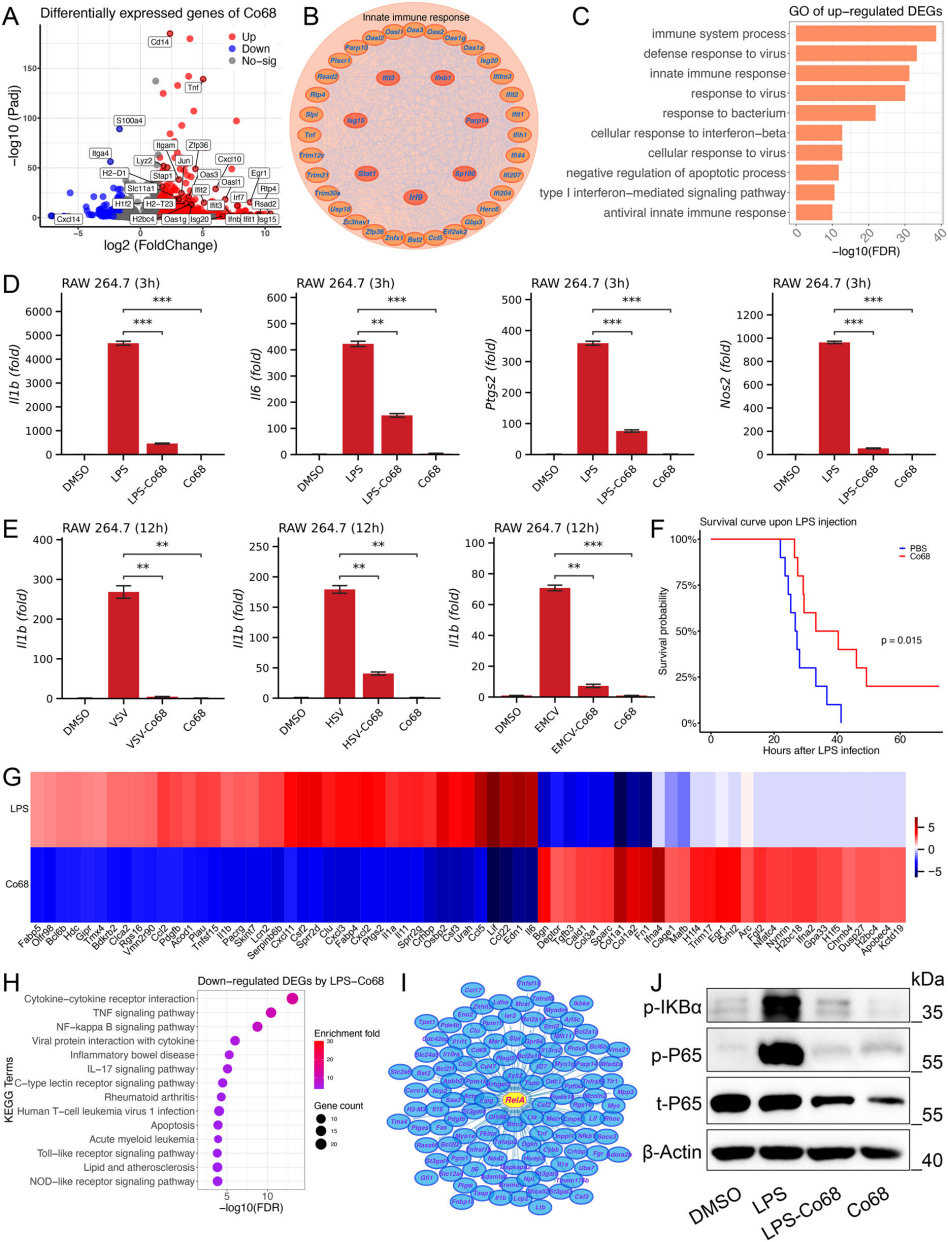
Co68 activates the innate immune response while suppressing inflammatory responses. (A) Volcano plot displaying log2‐transformed fold changes of differentially expressed genes (DEGs) between Co68‐ and DMSO‐treated RAW 264.7 cells. Red dots indicate upregulated DEGs in the Co68 group, and blue dots represent downregulated DEGs. (B) Protein‐protein interaction (PPI) network analysis of upregulated DEGs in Co68‐treated RAW 264.7 cells, highlighting key genes involved in the innate immune response. (C) Gene Ontology (GO) enrichment analysis of upregulated DEGs in Co68‐treated RAW 264.7 cells, revealing significant biological processes related to the innate immune response. (D) Co68 treatment reduces the expression of pro‐inflammatory cytokines (*Il1b*, *Il6*) and inflammatory mediators (*Ptgs2*, *Nos2*) in RAW 264.7 cells following lipopolysaccharide (LPS) stimulation. (E) Co68 suppresses *Il1b* expression induced by vesicular stomatitis virus (VSV, multiplicity of infection [MOI] = 0.1), herpes simplex virus 1 (HSV‐1, F strain, MOI = 0.5), and encephalomyocarditis virus (EMCV, MOI = 0.1) in RAW 264.7 cells. (F) Co68 improves survival rates in LPS‐challenged mice (*n* = 20, 20 mg/kg). (G) Heatmap illustrating the differential gene expression patterns between LPS‐ and Co68‐treated RAW 264.7 cells. (H) Kyoto Encyclopedia of Genes and Genomes (KEGG) analysis of downregulated DEGs in the LPS‐Co68 group compared to the LPS group, highlighting enriched inflammation‐related pathways. (I) Transcription factor enrichment analysis (TFEA) of downregulated DEGs in the LPS‐Co68 group compared to the LPS group identifies RelA as a potential key regulator. Yellow nodes represent transcription factors, and blue nodes represent downregulated genes by Co68. (J) Western blot analysis showing that Co68 inhibits LPS‐induced phosphorylation of P65 and IκBα, key regulators of the NF‐κB pathway. RT‐qPCR data are presented as means ± SEM from three independent experiments. Statistical significance was determined by one‐way ANOVA with Bonferroni's multiple comparisons test (D, E), or the log‐rank test (F). ^*^ *p* < 0.05, ^**^ *p* < 0.01, and ^***^ *p* < 0.001.

To elucidate the global transcriptional landscape regulated by Co68 in macrophages, we performed bulk RNA sequencing (RNA‐seq) on RAW 264.7 cells following a 3‐h treatment. Differential expression analysis revealed a total of 472 significantly upregulated and 153 downregulated genes in the Co68‐treated group relative to the DMSO control (Figure 3A). Consistent with its predicted dual‐functional profile, Co68 treatment prominently induced the expression of Type I Interferons (IFN‐I), interferon‐stimulated genes (ISGs), and major histocompatibility complex (MHC) molecules. Specifically, key immunomodulatory genes, including *Ifna2*, *Ifna4*, *Ifnb1*, *Oas1a*, *Rsad2*, *Ifit3*, *Cxcl10*, *Isg15*, *Isg20*, *H2‐D1*, *H2‐K1*, and *H2‐T23*, exhibited marked upregulation (Figure S3A). Protein‐protein interaction (PPI) network analysis via the STRING database was employed to decode the functional connectivity of these differentially expressed genes (DEGs). The most prominent clusters within the upregulated PPI network were predominantly associated with innate immune effector responses, the orchestration of IFN‐I production, and the restriction of viral genome replication (Figure 3B and Figure S3B,C), reinforcing the role of Co68 as a potent innate immune agonist. Gene Ontology (GO) enrichment analysis further corroborated these findings, showing that upregulated transcripts were highly enriched in pathways related to antiviral defense, innate immune activation, and IFN‐I‐mediated signaling (Figure 3C). In contrast, downregulated genes were primarily mapped to biological processes such as positive regulation of cell migration, apoptotic signaling, and ubiquitin‐dependent endocytosis (Figure S3D). Mechanistically, immunoblotting confirmed that Co68 treatment induced significant phosphorylation of TBK1 and IRF3, validating the activation of the canonical TBK1‐IRF3 axis essential for IFN‐I induction (Figure S3E).

The anti‐inflammatory efficacy of Co68 was subsequently evaluated by quantifying the expression of pivotal pro‐inflammatory mediators, including *Il1b*, *Il6*, *Nos2*, and *Ptgs2*. Co68 treatment significantly attenuated the lipopolysaccharide (LPS)‐induced upregulation of *Il1a*, *Il1b*, *Il6*, *Nos2*, *Ptgs2*, *Csf1*, *Csf2*, and *Csf3* (Figure 3D and Figure S3F). Intriguingly, while most pro‐inflammatory cytokines were suppressed, Co68 synergistically enhanced the production of *Tnf* in the presence of LPS, a phenomenon also observed for *Ifnb1*, *Cxcl10*, and *Isg15* (Figure S3G). Beyond LPS stimulation, Co68 demonstrated broad‐spectrum anti‐inflammatory activity by suppressing *Il1b* expression induced by various viral challenges, including VSV, HSV, and EMCV (Figure 3E). In a murine model of endotoxemia, Co68 administration markedly improved survival rates following a lethal intraperitoneal dose of LPS (Figure 3F). Transcriptomic profiling of the Co68+LPS group further revealed that Co68 uniquely reconfigures the inflammatory transcriptome. While LPS primarily drove an inflammatory gene signature, Co68 skewed the profile toward IFN‐I signaling and selectively induced several histone‐coding genes, such as *H1f5*, *H2bc4*, and *H2bc18* (Figure 3G), suggesting a specific role for Co68 in epigenetic or transcriptional regulation.

Kyoto Encyclopedia of Genes and Genomes (KEGG) pathway analysis of genes downregulated in the LPS‐Co68 group compared to the LPS‐only group identified the significant suppression of the NF‐κB signaling pathway, along with pathways associated with rheumatoid arthritis and Toll‐like/NOD‐like receptor signaling (Figure 3H). Gene Set Enrichment Analysis (GSEA) further substantiated this anti‐inflammatory phenotype, showing a profound negative enrichment of the “INFLAMMATORY_RESPONSE” hallmark (Figure S3H). Transcription factor enrichment analysis using the iRegulon framework identified the NF‐κB subunit RELA (P65) as a primary target of Co68‐mediated suppression (Figure 3I). Consistent with these in silico predictions, biochemical assays demonstrated that Co68 effectively inhibited the LPS‐induced phosphorylation and subsequent degradation of IκB‐α, as well as the phosphorylation of P65 and the expression of downstream effectors iNOS and Cox2 (Figure 3J and Figure S3I). Collectively, these findings establish that Co68 is a bifunctional therapeutic agent that robustly activates IFN‐I signaling while broadly antagonizing NF‐κB‐driven inflammatory responses.

### 2.4 Co68 Demonstrates Potent Antitumor Activity in the Pan02 Model

**FIGURE 4:**
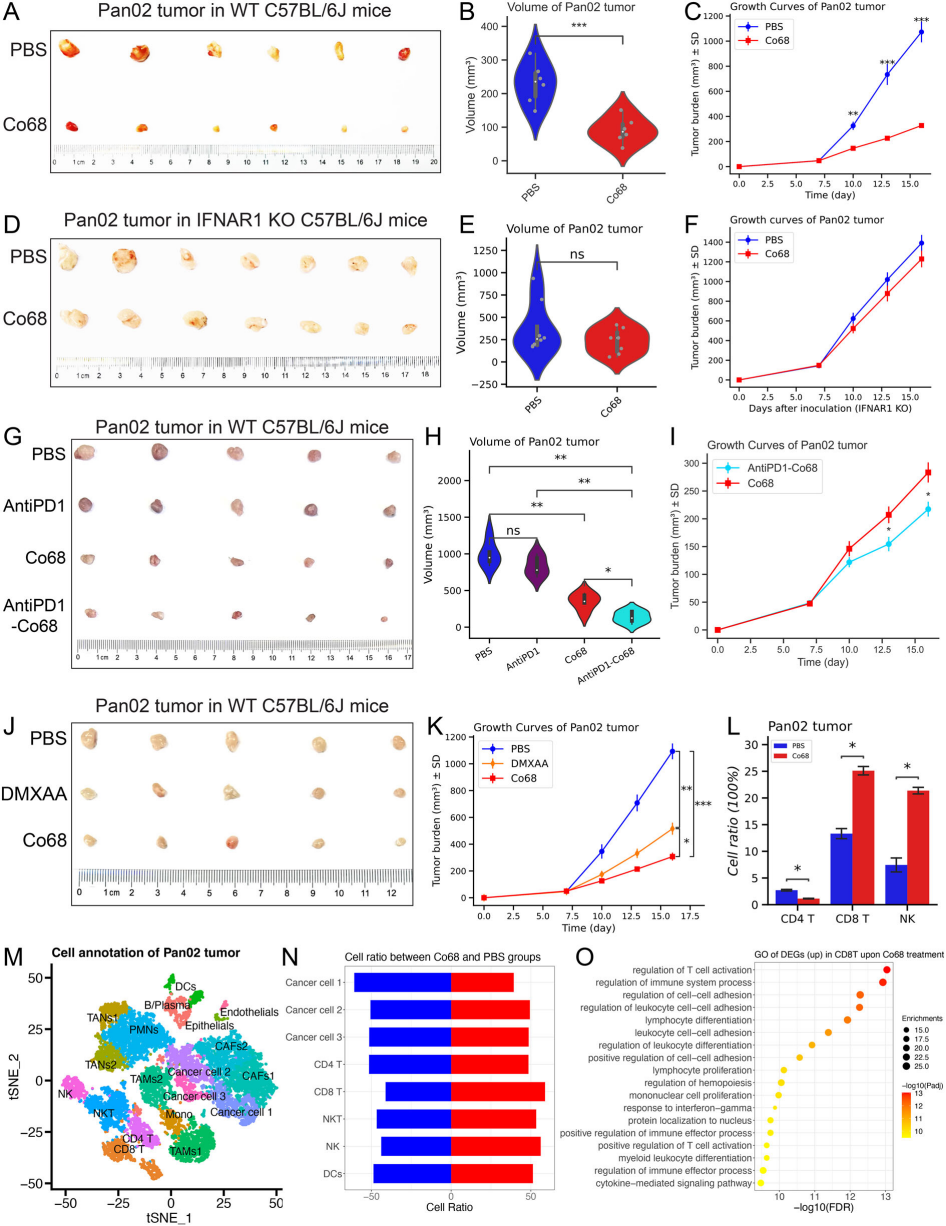
Co68 treatment inhibits the growth of the pancreatic cancer tumor (Pan02) in mice. (A, B, C) Representative images (A), quantification of tumor size (B), and growth curves (C) of Pan02 tumors in wild‐type (WT) mice (*n* = 6 per group) treated with Co68 (25 mg/kg, i.p.) or PBS via intraperitoneal (i.p.) injection every other day for 2 weeks. (D, E, F) Representative images (D), quantification of tumor size (E), and growth curves (F) of Pan02 tumors in IFNAR1 knockout (KO) mice (*n* = 7 per group) treated with Co68 (25 mg/kg, i.p.) or PBS via i.p. injection every other day for 2 weeks. (G) Tumor images of subcutaneous Pan02 implants in mice treated with isotype control antibody (200 µg/mouse, i.p.), Co68 (25 mg/kg, i.p.), anti‐PD‐1 antibody (200 µg/mouse, i.p.), or Co68 combined with anti‐PD‐1 antibody (*n* = 5 per group). (H) Quantification of tumor size of subcutaneous Pan02 implants in the same treatment groups as in panel G. (I) Comparison of growth curves for subcutaneous Pan02 tumors between the Co68 and AntiPD1‐Co68 treatment groups, as in panel J. (J) Tumor images of subcutaneous Pan02 implants in mice treated with DMXAA (25 mg/kg, i.p.), Co68 (25 mg/kg, i.p.), or solvent control (*n* = 5 per group) via i.p. injection every other day for 2 weeks. (K) Representative growth curves of subcutaneous Pan02 tumors in the same treatment groups as in panel J via i.p. injection every other day for 2 weeks. (L) Flow cytometry analysis of CD4 T cells, CD8 T cells, and NK cells in Pan02 tumors, showing increased cytotoxic immune cells in Co68‐treated tumors compared to the PBS group. (M) t‐SNE plot illustrating cell population clustering and annotation in Pan02 tumor samples treated with PBS or Co68, with clusters color‐coded by cell type. (N) Bar chart comparing the proportions of major cell types between Co68‐ and PBS‐treated groups in Pan02 tumors. (O) Gene Ontology (GO) enrichment analysis of upregulated differentially expressed genes (DEGs) in CD8^+^ T cells following Co68 treatment compared to PBS. Data were presented as means ± SEM for the indicated number of mice per group. Data are representative of three independent experiments. Statistical significance was determined by one‐way ANOVA with Bonferroni's multiple comparisons test. *ns*, not significant, *p* > 0.05; ^*^ *p* < 0.05; ^**^ *p* < 0.01; ^***^ *p* < 0.001.

Given the dual capacity of Co68 to robustly activate IFN‐I signaling and suppress macrophage‐mediated inflammation, we investigated whether Co68 could reprogram tumor‐associated macrophages (TAMs) and enhance antitumor immunity within the pancreatic cancer tumor microenvironment (TME). Utilizing a murine Pan02 pancreatic cancer model, we observed that Co68 administration significantly restricted tumor progression (Figure 4A–C and Figure S4A). Mechanistically, this antitumor efficacy was intrinsically linked to IFN‐I signaling, as the therapeutic benefit of Co68 was completely abrogated in *Ifnar1* knockout (KO) mice (Figure 4D–F and Figure S4B). In alignment with previous reports, Pan02 tumors exhibited a classic “cold” phenotype, characterized by minimal responsiveness to anti‐PD‐1 monotherapy (Figure 4G,H and Figure S4C). However, the combination of Co68 and anti‐PD‐1 antibody synergistically inhibited tumor growth, significantly outperforming either monotherapy (Figure 4G–I). Notably, while the STING agonist DMXAA demonstrated potent antitumor activity in the Pan02 model (Figure S4D–F), Co68 exhibited a more pronounced therapeutic effect (Figure 4J,K). We hypothesize that this superior efficacy stems from the unique anti‐inflammatory properties of Co68, which are absent in DMXAA. Furthermore, Co68 demonstrated robust antitumor activity in the MC38 colon carcinoma model (Figure S4G–I), suggesting its broad‐spectrum therapeutic potential across diverse histological malignancies.

Flow cytometric analysis corroborated these findings, revealing a substantial increase in infiltration of CD8^+^ T cells and NK cells, along with a reduction in CD4^+^ T cells in the Pan02 TME following Co68 treatment (Figure 4L). To decipher the underlying mechanisms and compare the efficacy of Co68 with DMXAA at single‐cell resolution, we performed single‐cell RNA sequencing (scRNA‐seq) on Pan02 tumors. Unsupervised clustering via t‐distributed Stochastic Neighbor Embedding (t‐SNE) identified 25 distinct subpopulations (Figure S4J). Comprehensive annotation based on cluster‐specific marker genes revealed a heterogeneous TME comprising malignant cells, cancer‐associated fibroblasts (CAFs), TAMs, tumor‐associated neutrophils (TANs), T cells, B cells, NK cells, dendritic cells (DCs), and endothelial/epithelial cells (Figure S4K).

The TME was predominantly composed of CAFs, with TAMs constituting the most abundant immune lineage (Figure 4M), consistent with the highly desmoplastic nature of pancreatic adenocarcinoma. Comparative analysis of immune cell proportions revealed that Co68 treatment significantly enriched the populations of CD8^+^ T cells and NK cells while markedly depleting the “Cancer Cell 1” subpopulation (Figure 4N), validating our flow cytometry data. Differential gene expression analysis indicated that CD8^+^ T cells in the Co68‐treated group exhibited a robust activation signature, characterized by the upregulation of genes involved in lymphocyte differentiation, proliferation, and *Ifng*‐mediated signaling (Figure 4O). Collectively, these data suggest that Co68 not only increases the numerical density of effector lymphocytes within the pancreatic TME but also enhances their functional activation, thereby fortifying the host's antitumor immune response.

### 2.5 Co68 Reprograms TAMs Within the Pancreatic Tumor Microenvironment

**FIGURE 5:**
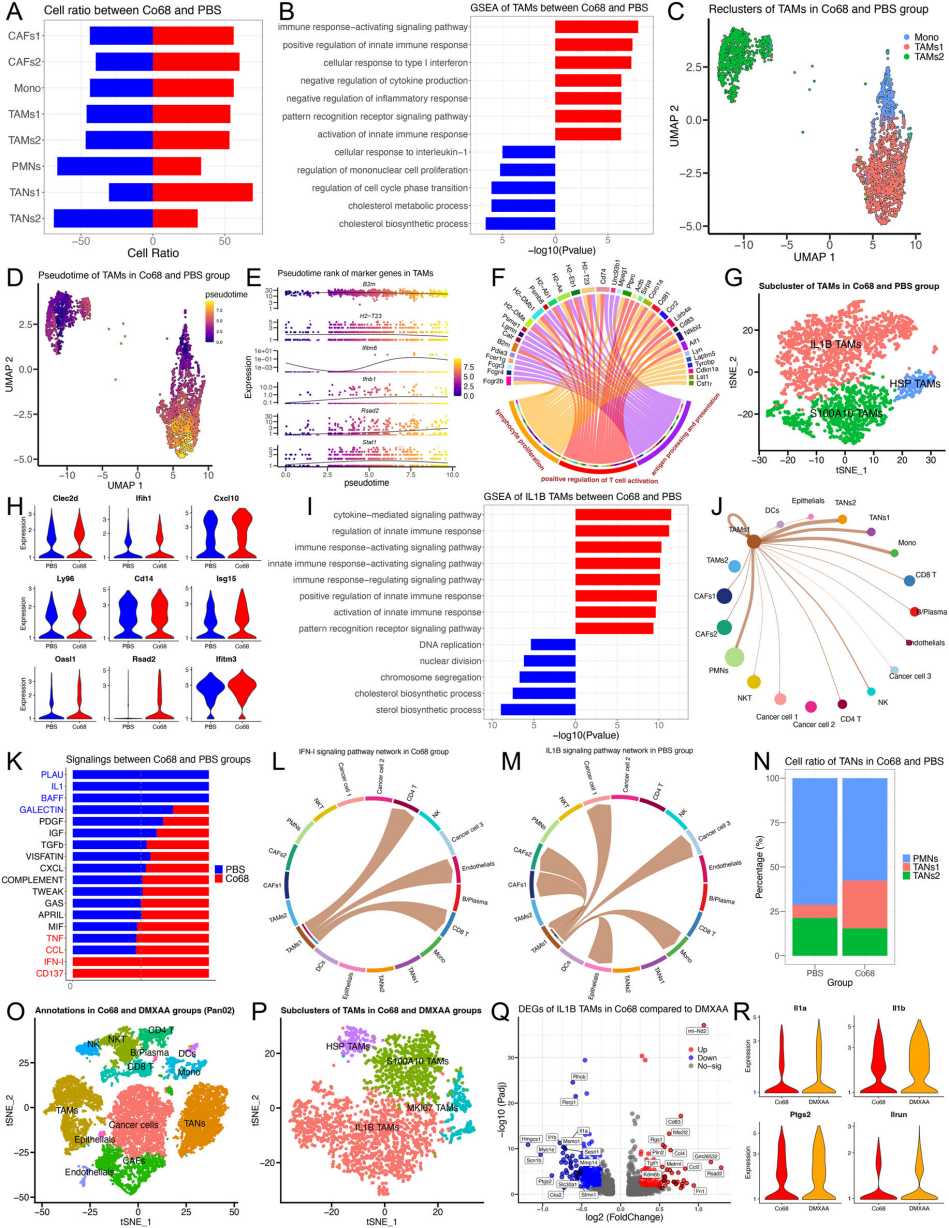
Co68 reprograms tumor‐associated macrophages (TAMs) within the tumor microenvironment (TME) in the Pan02 model. (A) Proportional distribution of tumor‐associated cell types in Pan02 tumors. (B) Gene set enrichment analysis (GSEA) of tumor‐associated macrophages (TAMs), showing enriched innate immune pathways in Co68‐treated tumors compared to PBS. (C) UMAP visualization showing tumor‐associated macrophages (TAMs) reclustering into three subpopulations in Co68‐ and PBS‐treated groups for downstream pseudotime analysis. (D) Pseudotime trajectory of TAMs in Co68‐ and PBS‐treated groups visualized by UMAP, with differentiation states indicated by a purple‐to‐yellow gradient. (E) Expression patterns of marker genes along the pseudotime trajectory of TAMs, with colors representing expression intensity. (F) Chord diagram showing associations between marker genes and key biological processes in the yellow color cluster at the end of the pseudotime trajectory analysis. Line thickness represents the strength of the association. (G) t‐SNE plot of TAM subclusters (IL1B TAMs, S100A10 TAMs, HSP TAMs) in Co68‐ and PBS‐treated groups. (H) Violin plots showing the expression of *Clec2d*, *Ifih1*, *Cxcl10*, *Ly96*, *Cd14*, *Isg15*, *Oasl1*, *Rsad2*, and *Ifitm3* in IL1B TAMs, which were significantly upregulated in Co68‐treated groups compared to PBS. (I) GSEA of IL1B TAMs comparing Co68 and PBS treatment groups, highlighting enriched pathways related to IFN‐I signaling in the Co68 group. (J) The interaction network of TAMs with other cell types in the tumor microenvironment shows predicted interactions. Line thickness indicates the strength of the interactions. (K) Bar plot comparing signaling pathways enriched in Co68‐ and PBS‐treated groups in IL1B TAMs. Blue bars represent pathways enriched in PBS, while red bars represent pathways enriched in Co68. (L, M) Chord diagrams representing IFN‐I (M) and IL1B signaling pathway (N) networks in Co68‐treated (M) and PBS‐treated (N) groups, respectively. Chord width reflects interaction strength between cell populations. (N) Comparison of distribution of TAN subpopulations (PMNs, TANs1, and TANs2) between Co68 and PBS groups. (O) t‐SNE plot illustrating cell type annotations in Pan02 tumors from Co68‐ and DMXAA‐treated groups. (P) t‐SNE plot showing four TAM subclusters (IL1B TAMs, S100A10 TAMs, MKI67 TAMs, and HSP TAMs) in Pan02 tumors from Co68‐ and DMXAA‐treated groups. (Q) Volcano plot of DEGs in IL1B TAMs comparing Co68‐ and DMXAA‐treated groups. Upregulated genes in the Co68 group are shown in red, downregulated genes in the Co68 group in blue, and non‐significant genes in gray. (R) Violin plots comparing the expression of inflammation‐related genes (*Il1a*, *Il1b*, *Ptgs2*, and *Ilrun*) between Co68‐ and DMXAA‐treated groups.

Intriguingly, scRNA‐seq analysis revealed that Co68 treatment significantly expanded the proportions of monocytes, tumor‐associated macrophages (TAMs), and the TANs1 neutrophil subpopulation compared to the PBS control (Figure 5A). Given the pivotal role of TAM‐derived *Il1b* and cancer cell‐derived PGE2 in driving pancreatic cancer progression and metastasis [5, 10, 11, 33], we focused on the phenotypic evolution of the TAM compartment. Gene set enrichment analysis (GSEA) demonstrated that Co68‐treated TAMs showed a robust induction of pathways associated with innate immune activation, Type I Interferon signaling, and pattern recognition receptor (PRR) signaling, coupled with significant negative regulation of inflammatory responses (Figure 5B). In contrast, TAMs from the control group remained enriched in interleukin‐1 (IL‐1) signaling and cholesterol metabolic processes (Figure 5B).

Pseudotime trajectory analysis identified the TAMs1 subpopulation as the primary focal point of Co68‐mediated reprogramming (Figure 5C–E). Detailed transcriptomic profiling of the TAMs1, Mono, and TAMs2 clusters revealed that TAMs1 exhibited a distinct activation signature, including the upregulation of genes involved in T cell priming, antigen processing/presentation, and lymphocyte proliferation (Figure 5F). Based on lineage‐specific markers, we redefined the TAMs1, Mono, and TAMs2 clusters as *Il1b* TAMs, HSP TAMs, and *S100a10* TAMs, respectively, with *Il1b* TAMs representing the highly Co68‐responsive effector population (Figure 5G). Further analysis of this subpopulation revealed the marked upregulation of critical PRRs and interferon‐stimulated genes (ISGs), such as *Clec2d*, *Ifih1*, *Ly96*, *Cd14*, *Ifitm3*, *Cxcl10*, *Isg15*, *Oasl1*, and *Rsad2* (Figure 5H). GSEA confirmed that Co68‐induced genes in *Il1b* TAMs were predominantly mapped to innate immune activation, while downregulated transcripts were associated with DNA replication and cholesterol biosynthesis (Figure 5I).

Intercellular communication analysis using the CellChat framework identified potent interactomes between *Il1b* TAMs and the “Cancer Cell 1” population, the latter of which was significantly depleted following Co68 treatment (Figure 5J and Figure S5A,B). These interactions were notably more robust than those observed for the *S100a10* TAM or Monocyte clusters (Figure S5C,D). Specifically, Co68 treatment selectively fortified IFN‐I‐mediated communication between *Il1b* TAMs and effector populations, including CD4^+^ T cells, CD8^+^ T cells, and endothelial cells (Figure 5K,L). Conversely, control *Il1b* TAMs maintained strong IL‐1‐mediated signaling with CAFs and malignant cells (Figure 5K,M). We also noted sustained *Spp1*‐mediated communication across diverse TME populations (Figure S5E), consistent with its established role in pancreatic cancer stroma [34, 35].

Beyond the macrophage compartment, Co68 treatment markedly altered the neutrophil landscape, favoring the expansion of the TANs1 subpopulation over TANs2 (Figure 5N and Figure S5F). TANs1 exhibited a robust ISG‐high signature, including the upregulation of *Ifit1*, *Ifit2*, *Ifit3*, *Rsad2*, *Parp14*, *Isg20*, *Irf7*, *Ifi204*, and *Ifi47* (Figure S5G). Functional enrichment associated these ISG^+^ TANs1 with antiviral‐like innate responses and *Ifnb1* signaling (Figure S5H), reinforcing the concept that IFN‐I‐polarized neutrophils contribute significantly to the Co68‐mediated antitumor effect. To further delineate the superior therapeutic index of Co68 over DMXAA, we performed comparative scRNA‐seq analysis. The two agonists elicited distinct shifts in cell subpopulation ratios (Figure 5O and Figure S5I), diverging from the baseline PBS‐treated TME (Figure 4M). Sub‐clustering of TAMs identified four lineages: *Il1b* TAMs, *S100a10* TAMs, HSP TAMs, and *Mki67* TAMs (Figure 5P and Figure S5J). Notably, Co68 treatment induced a significantly more potent IFN‐I response in *Il1b* TAMs compared to DMXAA, characterized by higher expression of *Il1rn*, *Oasl1*, *Rsad2*, *Tnf*, *Cxcl10*, and *Isg15* (Figure 5Q,R and Figure S5L). Crucially, Co68 uniquely suppressed pro‐inflammatory genes, including *Il1a*, *Il1b*, and *Ptgs2* (Figure 5R), a dual‐modulatory effect that DMXAA failed to achieve. GSEA confirmed that Co68‐treated *Il1b* TAMs were enriched in immune activation and TNF production pathways (Figure S5K). Collectively, these findings establish that Co68 orchestrates superior antitumor immunity by uncoupling IFN‐I activation from IL‐1‐driven inflammation, thereby reprogramming the myeloid landscape more effectively than conventional STING agonists.

### 2.6 Co68 Activates Type I Interferon via a Novel Binding Mode With TLR4

**FIGURE 6:**
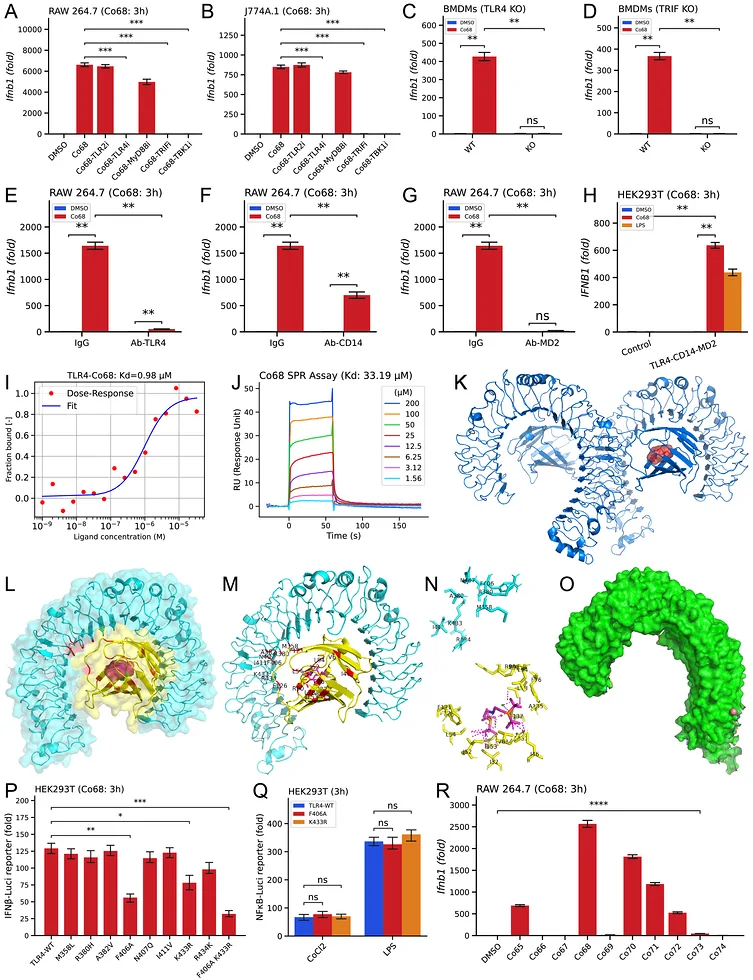
Co68 activates IFN‐I signaling by directly targeting TLR4. (A, B) Co68‐induced *Ifnb1* expression was significantly reduced in RAW 264.7 and J774A.1 macrophages following inhibition of the Toll‐like receptor 4 (TLR4) signaling pathway, when using inhibitors of TLR4, TRIF, and TBK1. (C, D) Co68 failed to induce *Ifnb1* expression in bone marrow‐derived macrophages (BMDMs) from TLR4 knockout (KO) or TRIF KO mice. (E–G) Neutralizing antibodies targeting TLR4, CD14, or MD2 significantly suppressed Co68‐induced *Ifnb1* expression in RAW 264.7 cells. Cells were pretreated with IgG isotype control or specific neutralizing antibodies for 48 h, followed by Co68 treatment for 3 h. (H) Co‐transfection of TLR4, CD14, and MD2 plasmids enabled Co68‐induced *Ifnb1* expression in HEK293T cells. (I) Dose‐response curve from microscale thermophoresis (MST) showing a dissociation constant (K*d*) of 0.98 µm for the interaction between Co68 and TLR4. (J) Surface plasmon resonance (SPR) analysis of Co68 binding to TLR4, with a real‐time binding curve and a calculated K*d* of 33.19 µm. (K) Molecular docking revealed that Co68 resides in the MD2 hydrophobic pocket of the TLR4‐MD2 complex. (L) Co68 was predicted to interact with both TLR4 and MD2 simultaneously (shown in red). (M) Amino acid sites where Co68 may interact with TLR4 and MD2. (N) Hydrogen bonds formed by Co68 atoms interacting with MD2 amino acids. (O) AlphaFold3‐predicted sites for direct interaction between Co ions and TLR4. (P) TLR4 mutations alter Co68‐induced IFNβ‐Luciferase reporter activity, highlighting critical residues for functional interaction. (Q) Mutations in TLR4 did not significantly impact NF‐κB Luciferase reporter activity in response to CoCl2 or lipopolysaccharide (LPS) treatment. (R) Comparison of Co68 and its nine analogs in RAW 264.7 macrophages demonstrates differential induction of *Ifnb1* expression. RT‐qPCR data and Luciferase assays were presented as means ± SEM from three independent experiments. Statistical significance was determined by one‐way ANOVA with Bonferroni's multiple comparisons test or paired‐samples *t*‐test. ^*^ *p* < 0.05, ^**^ *p* < 0.01, and ^***^ *p* < 0.001.

To identify the pattern recognition receptor (PRR) responsible for Co68‐mediated interferon activation, we employed a systematic approach utilizing pharmacological inhibitors and genetic knockout (KO) models. Inhibition of TLR4, TRIF, or the TBK1/IKKε complex significantly attenuated Co68‐induced *Ifnb1* expression in both RAW 264.7 and J774A.1 cells (Figure 6A,B). These findings were further validated using bone marrow‐derived macrophages (BMDMs) from *Tlr4* ^−^/^−^, *Trif* ^−^/^−^, and *Irf3* ^−^/^−^ mice (Figure 6C,D and Figure S6A), collectively confirming that Co68 triggers *Ifnb1* expression through the canonical TLR4‐TRIF‐TBK1‐IRF3 signaling axis.

While LPS‐mediated TLR4 activation typically requires the co‐receptors CD14 and MD2, previous reports suggest that inorganic Co^2^^+^ can directly activate TLR4 independently of these molecules. To determine the co‐receptor requirements for Co68, we utilized specific neutralizing antibodies. Blocking TLR4, CD14, or MD2 significantly reduced the production of *Ifnb1* and *Isg15* (Figure 6E–G and Figure S6B–D). Notably, anti‐TLR4 and anti‐MD2 antibodies nearly abolished the induction of *Ifnb1*, suggesting that MD2 plays a more pivotal role than CD14 in Co68‐mediated IFN‐I activation. This was further corroborated in HEK293T cells, where ectopic expression of TLR4, CD14, and MD2 rendered the cells highly responsive to Co68 treatment (Figure 6H and Figure S6E). To characterize the physical interaction between Co68 and TLR4, we performed microscale thermophoresis (MST) and surface plasmon resonance (SPR) assays. MST analysis yielded a dissociation constant ($K_d$) of 0.98 µm for the Co68‐TLR4 interaction, indicating a higher binding affinity than that of LPS ($K_d$ = 4.37 µm) (Figure 6I and Figure S6F). Similarly, SPR analysis revealed a $K_d$ of 31.71 µm for Co68, compared to 147.64 µm for LPS (Figure 6J and Figure S6G–I). These data demonstrate that Co68 binds directly to TLR4 with greater stability than LPS, suggesting a non‐canonical binding mechanism.

We further explored this unique interaction using computational docking with AlphaFold3 and AutoDock Vina. The crystal structure of Co68 was first visualized (Figure S6J) before docking into the TLR4‐MD2 complex. The most stable binding configuration involved Co68 embedding into the hydrophobic pocket of MD2 with a binding energy < −7 kcal/mol (Figure 6K and Figure S6K). Co68 engaged in extensive contacts with both TLR4 and MD2, with a more pronounced interaction observed with the latter (Figure 6L and Figure S6L). Specifically, PyMOL analysis identified eight key residues in TLR4—ARG434, MET358, ARG380, ALA382, PHE406, ASN407, ILE411, and LYS433—that interact with Co68 (Figure 6M and Figure S6M,N). This binding fingerprint is distinct from the reported LPS‐binding residues (e.g., ARG264, LYS341). Furthermore, chemical bonding analysis predicted hydrogen bonds between Co68 and MD2 residues ILE32, ILE46, ILE52, LEU54, VAL61, LEU78, CYS133, and ILE153, a pattern markedly different from the AlphaFold3‐predicted Co^2^^+^ binding mode (Figure 6N,O and Figure S6O).

To experimentally validate this predicted interface, we generated TLR4 variants with point mutations at the eight identified residues. Luciferase reporter assays revealed that F406A and K433R mutations significantly impaired Co68‐induced *Ifnb1* production, with the double mutation exhibiting the most profound effect (Figure 6P). Crucially, these mutations did not affect NF‐κB activation induced by LPS or CoCl_2_, confirming that Co68 utilizes a unique structural interface to selectively drive IFN‐I signaling (Figure 6Q). Finally, structure‐activity relationship (SAR) analysis of nine Co68 analogs (Figure S6P) revealed that the PNP pincer scaffold, the tertiary butyl groups on the phosphate radical, the chloride ion coordination to the Co^2^^+^ center, and the hydrogen atom on the nitrogen bridge are all indispensable for *Ifnb1* induction (Figure 6R). Analogs that retained potent *Ifnb1* induction also exhibited stable TLR4 binding in SPR assays (Figure S7A–F). Consistent with these mechanistic insights, the in vivo antitumor activity of Co68 was completely lost in *Tlr4* ^−^/^−^ and *Irf3* ^−^/^−^ mice (Figure S7G–L). Collectively, these findings establish that Co68 activates a selective IFN‐I response through a unique binding mode within the TLR4‐MD2 complex, distinguishing it from both LPS and inorganic cobalt. Together, these structure–activity data indicate that Co68's immunomodulatory activity arises from a synergistic, coordination‐dependent interplay between the cobalt center and the PNP ligand, rather than from either component alone (further discussed below).

### 2.7 Co68 Inhibits Inflammatory Responses Through the Non‐Canonical Activation of STAT1

**FIGURE 7:**
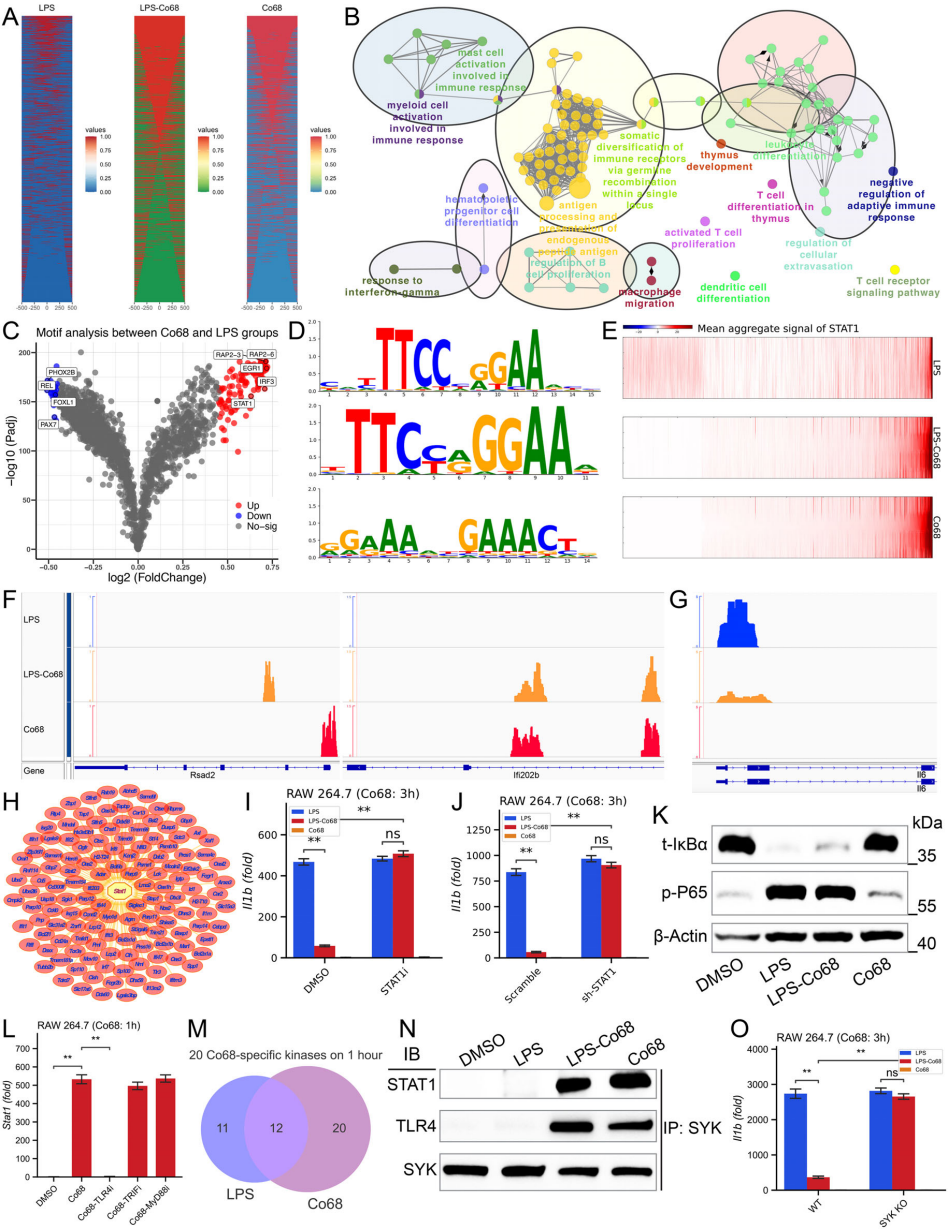
Co68 abolishes the inflammatory response by nonclassically activating STAT1. (A) Distribution of peaks around the transcription start site (TSS) in the LPS, LPS‐Co68, and Co68 groups compared to the DMSO group. (B) Gene Ontology (GO) annotation of differentially accessible regions (DARs) near genes involved in immune response‐related pathways. (C) Volcano plot showing differential enrichments of transcription factors (TFs) predicted by motif enrichment analysis between the Co68 and LPS treatment groups. (D) STAT1 binding motifs derived from the JASPAR database. (E) Mean aggregate binding signal of STAT1 across LPS, LPS‐Co68, and Co68 groups compared to the DMSO group. (F, G) Peak tracks of *Rsad2*, *Ifi202b*, and *Il6* visualized in IGV software for the LPS, LPS‐Co68, and Co68 groups compared to the DMSO group. (H) Transcription factor enrichment analysis (TFEA) of upregulated differentially expressed genes (DEGs) in the Co68 group compared to the LPS group after 1 h of treatment in RAW 264.7 cells, identifying STAT1 as a potential transcription factor. Yellow nodes represent enriched transcription factors, while red nodes represent upregulated DEGs in Co68‐treated RAW 264.7 cells. (I) Inhibition of STAT1 reduced Co68‐induced anti‐inflammatory effects in LPS‐stimulated RAW 264.7 cells. (J) Knockdown of STAT1 abrogated the anti‐inflammatory effect of Co68 in LPS‐stimulated RAW 264.7 cells. (K) Western blot analysis showing that Co68 inhibited LPS‐induced phosphorylation of P65 and IκBα after knockdown of STAT1. (L) Inhibition of the TLR4 receptor significantly reduced Co68‐induced STAT1 expression in RAW 264.7 cells. (M) Venn diagram showing the overlap and unique kinases specific to LPS and Co68 treatments, identified by mass spectrometry after one hour of treatment with Co68 and LPS. (N) Co‐immunoprecipitation (CoIP) analysis of TLR4‐SYK and STAT1‐SYK interactions. (O) SYK knockout abrogated Co68's anti‐inflammatory effect in LPS‐stimulated RAW 264.7 cells. RT‐qPCR data were presented as means ± SEM from three independent experiments. Statistical significance was determined by one‐way ANOVA with Bonferroni's multiple comparisons test or paired‐samples t‐test. ^*^ *p* < 0.05, ^**^ *p* < 0.01, ^***^ *p* < 0.001.

As previously established, Co68 possesses broad‐spectrum anti‐inflammatory properties and selectively induces histone‐modifying gene expression. We hypothesized that these effects are mediated by specific Co68‐responsive transcription factors (TFs). To map the early‐stage chromatin accessibility landscape prior to the detectable induction of *Ifnb1*, we performed ATAC‐seq on RAW 264.7 cells treated with LPS, Co68, or their combination for 1 h. Even at this early kinetic point, Co68 exhibited a potent inhibitory effect on LPS‐induced signatures. ATAC‐seq identified 50165, 41324, and 33316 accessible peaks in the LPS, LPS+Co68, and Co68 groups, respectively (Figure S8A–C). Genomic annotation via ChIPseeker revealed that Co68 and LPS+Co68 treatments significantly enriched accessible peaks in promoter regions compared to LPS monotherapy (Figure S8D). Furthermore, Co68 treatment markedly increased the density of TF‐binding sites within 3 kb of transcription start sites (TSS), whereas LPS‐induced peaks were predominantly localized in distal intergenic regions (Figure S8D,E). Heatmap analysis confirmed a global shift in chromatin accessibility toward TSS‐proximal regions in Co68‐treated groups (Figure 7A). Functional annotation of genes associated with differentially accessible regions (DARs) between the LPS‐Co68 and LPS groups highlighted pathways involved in myeloid activation, T cell receptor signaling, and macrophage migration (Figure 7B).

To identify the TFs driving these chromatin changes, we performed TOBIAS footprinting analysis. As expected, the NF‐κB family member REL exhibited higher occupancy in the LPS‐treated group (Figure 7C and Figure S8H). Conversely, the Co68‐treated group showed significant enrichment of IRF3 and EGR1 motifs. Intriguingly, STAT1 occupancy was markedly elevated in Co68‐treated samples as early as 1 h post‐treatment, significantly preceding the secretion of *Ifnb1* and subsequent IFNAR signaling (Figure 7C). The consensus binding motif for STAT1 and its markedly higher occupancy in Co68‐treated cells are shown in Figure 7D,E and Figure S8F. Track visualization using IGV further substantiated these findings; Co68 treatment attenuated binding signals at pro‐inflammatory loci such as *Il6*, *Il1b*, and *Tnf* (Figure 7G and Figure S8J,K), while simultaneously enhancing accessibility at ISG loci including *Rsad2*, *Ifi202b*, *Usp18*, and *Ifit1* (Figure 7F and Figure S8I). This early‐stage activation was corroborated by RNA‐seq (Figure 7H and Figure S8L), suggesting that Co68‐induced STAT1 activation occurs independently of the canonical IFN‐I/IFNAR feedback loop.

To evaluate the functional necessity of this early STAT1 activation, we employed the pharmacological inhibitor fludarabine and *Stat1* shRNA. Both fludarabine treatment and *Stat1* knockdown completely abrogated the anti‐inflammatory efficacy of Co68 (Figure 7I–K and Figure S8N). To delineate the upstream regulators, we utilized pathway‐specific inhibitors; notably, only TLR4 inhibition—but not MyD88 or TRIF inhibition—suppressed early STAT1 activation, indicating a non‐canonical TLR4‐dependent, MyD88/TRIF‐independent mechanism (Figure 7L). To pinpoint the specific kinase bridging TLR4 and STAT1, we conducted mass spectrometry on TLR4‐immunoprecipitated (IP) complexes 1 h post‐treatment (Figure S8M). Among the 20 kinases specifically enriched in the Co68 group, we identified SYK, a tyrosine kinase known to phosphorylate STAT1 (Figure 7M). Co‐immunoprecipitation (Co‐IP) confirmed the formation of a TLR4‐SYK‐STAT1 complex specifically in Co68‐treated cells (Figure 7N). Finally, both *Syk* knockdown and CRISPR‐Cas9‐mediated *Syk* knockout (KO) abolished the anti‐inflammatory activity of Co68 (Figure 7O and Figure S8O–Q). Collectively, these results demonstrate that Co68 exerts broad‐spectrum anti‐inflammatory effects through a unique, early‐acting TLR4‐SYK‐STAT1 signaling axis.

## 3 Discussion

The central challenge in innate immune agonist development has been the inseparable coupling of beneficial IFN‐I activation with detrimental inflammatory toxicity [19, 36, 37, 38, 39]. This study addresses this longstanding bottleneck by demonstrating that these two outputs can be mechanistically uncoupled through rational molecular design. First, we developed Deep Learning for Innate Immunity Modulatory Potential (DLINP), a Transformer‐based discovery framework designed to identify small molecules capable of uncoupling beneficial IFN‐I induction from deleterious inflammatory signaling. This strategy led to the prioritization of Co68, a PNP‐pincer cobalt complex with dual immunomodulatory activity. Second, we show that Co68 functions as a biased TLR4‐MD2 agonist: it activates the TLR4‐TRIF‐IFN‐I axis while suppressing NF‐kB‐driven inflammation through an early TLR4‐SYK‐STAT1 pathway. This signaling bifurcation distinguishes Co68 from conventional innate immune agonists that often couple immune activation with inflammatory toxicity. Third, in a PD‐1‐resistant Pan02 pancreatic cancer model, Co68 reprogrammed the tumor microenvironment by disrupting the IL‐1β‐PGE2 inflammatory circuit, promoting ISG‐high myeloid states, and increasing infiltration by CD8^+^ T cells and NK cells. Together, these findings establish Co68 as a precision innate immune agonist that converts inflammatory immune activation into a therapeutically favorable antitumor response.

Beyond identifying a single lead compound, this work highlights the potential of Transformer‐based AI frameworks for mechanism‐oriented immunomodulator discovery. Unlike conventional virtual screening strategies that prioritize predefined binding affinity or general drug‐like properties, DLINP was designed to search for a functional immune phenotype: simultaneous enhancement of IFN‐I signaling and suppression of inflammatory activation. This phenotype‐guided strategy enabled the discovery of Co68, a PNP‐pincer cobalt complex with dual immunomodulatory activity that was not reproduced by either component alone. The PNP ligand did not activate IFN‐I signaling in RAW 264.7 macrophages, whereas cobalt chloride showed only weak inflammatory activity at high concentration and failed to induce early transcriptional remodeling. In contrast, the coordinated Co68 complex rapidly activated IFN‐I signaling at lower concentrations while retaining broad anti‐inflammatory activity against LPS‐ and virus‐induced inflammatory stimuli. Structure‐activity analyses further supported the importance of metal‐ligand coordination: modification of the nitrogen‐bound hydrogen in the PNP scaffold or replacement of chloride ligands markedly impaired Co68‐mediated IFN‐I activation. These findings suggest that the biological activity of Co68 arises from an emergent coordination‐dependent property rather than from simple additive effects of the ligand or cobalt salt. More broadly, this study illustrates how AI‐guided phenotypic screening can uncover chemically unconventional scaffolds with predefined immune‐signaling logic, providing a scalable route for developing next‐generation innate immune agonists with improved therapeutic indices.

Mechanistically, our findings extend the current understanding of biased TLR4 signaling. Classical TLR4 agonists, including LPS‐like ligands, typically activate both the TRIF‐IRF3 and MyD88‐NF‐kB branches, thereby coupling antiviral IFN‐I induction with inflammatory cytokine production. Among these, monophosphoryl lipid A (MPLA), a clinically used TRIF‐biased TLR4 agonist derived from lipid A, engages the hydrophobic pocket of MD2 through its acyl chains, and its reduced acylation relative to LPS is thought to favor TRIF‐dependent signaling over MyD88‐dependent inflammation. In contrast, Co68 is a compact, non‐lipid organometallic scaffold that, based on our docking and mutagenesis data, engages the TLR4‐MD2 complex through a distinct, non‐acyl binding interface. This suggests that biased TLR4 signaling can be achieved through structurally divergent chemotypes — lipid‐based partial acylation in the case of MPLA, vs. compact metal–ligand coordination in the case of Co68 — highlighting non‐lipid organometallic scaffolds as a previously underexplored strategy for designing biased TLR4 modulators [40, 41, 42, 43]. Using complementary pharmacological, genetic, and biophysical approaches, including MST and SPR, we show that Co68 directly engages the TLR4‐MD2 complex. AlphaFold‐based structural modeling, molecular docking, and point‐mutagenesis experiments further suggest that Co68 adopts a binding mode distinct from cobalt ions and canonical TLR4 agonists. This non‐canonical interaction provides a plausible basis for the biased signaling profile of Co68: preferential activation of the TLR4‐TRIF‐IRF3‐IFN‐I axis, together with suppression of inflammatory outputs through an early TLR4‐SYK‐STAT1 pathway. Although our mutagenesis studies support the proposed binding interface, future quantitative biophysical analyses of mutant TLR4–MD2 complexes and high‐resolution structural studies will be required to distinguish direct effects on ligand affinity from potential mutation‐induced conformational changes. Cryogenic electron microscopy (cryo‐EM) is currently being employed to gain detailed structural insights into the Co68‐TLR4 interaction. Thus, rather than acting as a conventional pan‐TLR4 activator, Co68 appears to rewire TLR4 signaling toward an interferon‐dominant, inflammation‐restrained state. This unique mechanism may explain certain clinical observations in cancer therapies. For instance, paclitaxel, a widely used chemotherapeutic agent, targets TLR4, but its albumin‐bound formulation (nab‐paclitaxel) has been linked to enhanced tumorigenesis in specific clinical settings [13, 15, 16]. This discrepancy may stem from structural differences in their binding modes to TLR4, resulting in divergent effects on immune signaling and inflammation.

A pivotal finding of this study is the identification of STAT1 as a critical transcription factor in Co68‐induced early activation, which is independent of IFN‐I signaling and essential for the compound's anti‐inflammatory effects. ATAC‐seq and transcription factor footprinting revealed a Co68‐specific chromatin accessibility program enriched for STAT1 motifs, and STAT1 activation occurred before detectable Ifnb1 induction, indicating that this response is not simply secondary to autocrine IFN‐I signaling. Genetic and pharmacological analyses further showed that TLR4 is required for Co68‐induced STAT1 activation, whereas MyD88 and TRIF are dispensable for this early event. Proteomic analysis nominated SYK as an upstream kinase, and co‐immunoprecipitation experiments showed that SYK associates with both TLR4 and STAT1 in the presence of Co68. These findings support a previously underappreciated TLR4‐SYK‐STAT1 signaling pathway that restrains NF‐kB‐dependent inflammatory outputs while allowing TRIF‐dependent IFN‐I activation [44, 45, 46]. The TLR4‐SYK‐STAT1 axis emerges as a promising therapeutic target for inflammatory diseases. Although the precise mechanisms by which STAT1 mediates its anti‐inflammatory effects remain to be fully understood, we propose that STAT1 activation may resemble the action of STAT3 in TLR4 signaling, where phosphorylation of serine 727 by downstream kinases such as TBK1 induces metabolic reprogramming and anti‐inflammatory effects [47]. Further studies are needed to validate this hypothesis and deepen our understanding of the signaling dynamics at play. Co68 provides dual therapeutic benefits by concurrently activating the TLR4‐TRIF and TLR4‐SYK‐STAT1 signaling pathways, thereby mitigating inflammation while enhancing antiviral and antitumor immunity.

Several limitations should be acknowledged. Although this study establishes the molecular mechanism of Co68 and demonstrates robust antitumor activity in murine and humanized models, additional validation will be required before clinical translation. In particular, patient‐derived xenograft models and broader human tumor systems will be important for assessing whether Co68‐mediated TME reprogramming extends across heterogeneous pancreatic cancer contexts and other immune‐refractory tumors. Comprehensive pharmacokinetic, biodistribution, and toxicological studies are also needed to define the exposure‐response relationship, therapeutic window, and long‐term safety profile of this organometallic complex. In addition, although MST, SPR, docking, and mutagenesis support direct engagement of the TLR4‐MD2 complex, high‐resolution structural characterization of the Co68‐TLR4‐MD2 complex by cryo‐electron microscopy will be important for defining the molecular basis of signaling bifurcation. Although single‐cell transcriptomics revealed coordinated remodeling of TAMs and cytotoxic lymphocytes following Co68 treatment, future spatial transcriptomic and multiplex immunofluorescence analyses will be important to define the spatial organization and cellular interactions underlying this immune remodeling. In addition, although scRNA‐seq identified a marked expansion of an ISG‐high TANs1 neutrophil subset following Co68 treatment, functional studies — such as neutrophil depletion — will be required to determine whether this population is causally required for Co68‐mediated antitumor efficacy, and such experiments are currently underway in our laboratory. Similarly, our conclusion that Co68 disrupts the IL‐1β–PGE2 communication axis between Il1b TAMs and cancer cells is based on CellChat‐inferred transcriptomic signatures; direct functional validation, such as co‐culture or receptor‐blockade experiments, will be needed to confirm whether this axis is mechanistically required for antitumor efficacy. Addressing these questions will facilitate the future development of precision innate immune agonists that retain antitumor IFN‐I activity while minimizing inflammatory toxicity.

In conclusion, Co68 represents a precision innate immune agonist that leverages a PNP‐pincer organometallic scaffold to uncouple IFN‐I‐mediated antitumor immunity from inflammatory toxicity. By engaging the TLR4‐MD2 complex in a biased manner, Co68 activates TRIF‐dependent IFN‐I signaling while restraining NF‐kB‐driven inflammation through the TLR4‐SYK‐STAT1 axis. This dual activity enables effective remodeling of immunosuppressive tumor microenvironments and enhances responsiveness to PD‐1 blockade. More broadly, our findings establish Co68 as a proof of concept for AI‐guided discovery of metal‐based immunomodulators and provide a framework for developing safer, precision‐targeted innate immune agonists for immune‐refractory cancers.

## 4 Materials and Methods

### 4.1 Reagents and Antibodies

Rabbit anti‐COX2 antibodies were sourced from Wanleibio. Antibodies targeting LY96/MD2 (catalog no. 11784‐1‐AP), iNOS (catalog no. 80517‐1‐RR), TBK1 (catalog no. 28397‐1‐AP), NF‐κB p65 (catalog no. 10745‐1‐AP), and Beta Tubulin (catalog no. 10094‐1‐AP) were obtained from Proteintech. Mouse monoclonal antibodies against Beta Actin (catalog no. 66009‐1‐Ig), TLR4 (catalog no. 66350‐1‐Ig), CD14 (catalog no. 60253‐1‐Ig), and the HA tag (catalog no. 51064‐2‐AP) were also purchased from Proteintech. Phosphorylation‐specific antibodies, including rabbit anti‐phospho‐NF‐κB p65 (Ser536) (93H1) (catalog no. 3033), anti‐phospho‐IκBα (Ser32) (14D4) (catalog no. 2859), anti‐phospho‐TBK1/NAK (Ser172) (D52C2) (catalog no. 5483), anti‐phospho‐IRF‐3 (Ser396) (D6O1M) (catalog no. 29047), as well as antibodies for IRF‐3 (D6I4C) (catalog no. 11904), Stat1 (D1K9Y) (catalog no. 14994), and Syk (D3Z1E) (catalog no. 13198), were obtained from Cell Signaling Technology. For secondary detection, horseradish peroxidase (HRP)‐conjugated goat anti‐rabbit IgG (H+L) (catalog no. SA00001‐2) and HRP‐conjugated goat anti‐mouse IgG (H+L) (catalog no. SA00001‐1) were both sourced from Proteintech. Flow cytometry antibodies included FITC‐conjugated anti‐mouse NK1.1 (catalog no. FITC‐65138‐25UG) and PE‐conjugated anti‐mouse CD8a (catalog no. PE‐65069), which were procured from Proteintech, while APC‐conjugated anti‐mouse CD4 (catalog no. E‐AB‐F1097UE) was obtained from Elabscience. Recombinant mouse GM‐CSF (catalog no. HY‐P7361) and anti‐mouse PD‐1 antibody (catalog no. HY‐P99144) were purchased from MedChemExpress. Recombinant human TLR4 protein (ECD, His Tag) was obtained from Sino Biological (catalog no. 10146‐H08B).

### 4.2 Cell Culture

The cell lines RAW264.7, J774A.1, HEK293T, THP‐1, and Vero were obtained from the American Type Culture Collection (ATCC). Bone marrow‐derived macrophages (BMDMs) were isolated from the femurs and tibiae of eight‐week‐old C57BL/6J mice. The bone marrow was cultured in the presence of granulocyte‐macrophage colony‐stimulating factor (GM‐CSF, RP01206, ABclonal) for 7 days to promote macrophage differentiation. Peritoneal macrophages (PMs) were similarly isolated from the peritoneal cavity and differentiated using GM‐CSF for 7 days. HEK293T, RAW264.7, J774A.1, THP‐1, Vero, and BMDMs were cultured in Dulbecco's Modified Eagle's Medium (DMEM; 03.1002C, EallBio), while PMs were maintained in RPMI 1640 (03.4001C, EallBio). All culture media were supplemented with 10% fetal bovine serum (FBS) and 1% penicillin‐streptomycin to support optimal cell growth and maintenance.

### 4.3 Virus Infection and Propagation

Cells at 70%–80% confluence were infected with the following viruses at the indicated multiplicities of infection (MOI): Vesicular Stomatitis Virus (VSV, MOI = 0.1), Herpes Simplex Virus 1 (HSV‐1, F strain, MOI = 0.5), and Encephalomyocarditis Virus (EMCV, MOI = 0.1). Virus stocks were prepared as follows: the VSV Indiana strain, kindly provided by Dr. J. Rose (Yale University), was propagated in Vero cells. The HSV‐1 strain 17, a gift from Dr. Zhengfan Jiang (Peking University), was cultured in Vero cells for viral propagation. The EMCV strain (ATCC VR‐129B) was acquired from ATCC and similarly propagated in Vero cells.

### 4.4 Mice and Construction of a Sepsis Model via LPS Injection

Wild‐type (WT) C57BL/6J mice were obtained from the Department of Laboratory Animal Science, Peking University Health Science Center. *Tlr4_−_/_−_*, *Irf3_−_/_−_*, and *Trif_−_/_−_* mice, all on a C57BL/6J background, were acquired from the Institute of Experimental Animals, Chinese Academy of Medical Sciences. The *Ifnar1_−_/_−_* mice were generously provided by Prof. Erol Fikrig (Yale University). Genotyping of these mice was performed using the following primers: *Tlr4* KO (forward: 5′‐TGCTCACACCATCATCAC‐3′; reverse: 5′‐CATGTACTAGGTTCGTCAGA‐3′), *Irf3* KO (forward: 5′‐TCGTGCTTTACGCTATGCCGCTCCCGATT‐3′; reverse: 5′‐GAACCTCGGAGTTATCCCGAAGG‐3′), and *Ifnar1* KO (forward: 5′‐CGAGGCGAAGTGGTTAAAAG‐3′; reverse: 5′‐AATTCGCCAATGACAAGACG‐3′). All animal experiments were carried out in accordance with the *Guide for the Care and Use of Laboratory Animals* provided by the Chinese Association for Laboratory Animal Science. The protocols were approved by the Animal Care Committee of Peking University Health Science Center (permit number: LA 2016240). Mice were housed under specific pathogen‐free (SPF) conditions at the Laboratory Animal Center of Peking University. Only male mice aged 6 to 8 weeks were used in the study. Lipopolysaccharide (LPS, HY‐D1056, MedChemExpress) was dissolved in sterile PBS (P1020, Solarbio Life Science) to a final concentration of 20 mg/kg body weight. A total of 20 WT C57BL/6J mice were injected intraperitoneally with LPS using sterile insulin syringes. Following LPS injection, a subset of mice (*n* = 10) received a 25 mg/kg dose of Co68 (200 µL in PBS) via intraperitoneal injection. The control group (*n* = 10) was treated with an equivalent volume of PBS. Survival times were monitored and recorded following injection.

### 4.5 Tumor Transplantation, Treatments, and Tissue Digestion

Mice were subcutaneously injected into the right flank with 5 × 10^5^ Pan02 cells suspended in 100 µL PBS, unless otherwise specified. Tumor growth was monitored daily, with tumor size measured every 1–2 days. Tumor volume was calculated using the formula: volume = length (mm) × width^2^ (mm^2^) × 0.5. Tumor‐bearing mice were treated with 25 mg/kg Co68 or DMXAA via intraperitoneal injection every 2 days. For immune checkpoint blockade, tumor‐bearing mice were administered 200 µg of anti‐mouse PD‐1 antibody (200 µL saline) via intraperitoneal injection on days 3, 7, and 11 following tumor inoculation. Control mice received 200 µL of rat IgG2a isotype (Clone 2A3, BioXCell) on the same schedule. Following euthanasia, tumors and the surrounding skin were excised. Tumors were minced and digested in RPMI 1640 medium containing 0.5 mg/mL collagenase D (11088866001, Roche) and 0.1 mg/mL DNase I (DN25‐100G, Sigma–Aldrich) at 37°C for 1 h. The resulting cell mixture was then filtered through a 70‐µm cell strainer to obtain a single‐cell suspension, which was subsequently transferred to flow cytometry tubes for further analysis.

### 4.6 RNA Extraction, Reverse Transcription, and Real‐Time Quantitative PCR

RNA extraction from cells or tissues following various treatments or infections was performed using TRIzol reagent (TIANGEN, A0123A01). The purified RNA was then reverse transcribed into cDNA using HiScript II RT SuperMix (Vazyme, R223‐01). Quantification of target gene expression was carried out using SYBR Green qMix (Vazyme, Q311) in a quantitative reverse transcription PCR (RT‐qPCR) assay. The relative expression levels of target mRNAs were normalized to the housekeeping gene *Gapdh*. Detailed primer sequences used in this study are provided in Table S1.

### 4.7 Luciferase Assay

Cells were seeded into 6‐well plates and cultured to 70%–80% confluence. Transient transfection was performed using Lipofectamine 2000 (Thermo Fisher) according to the manufacturer's instructions. After 24 h of transfection, the cells were treated with Co68, CoCl_2_, or LPS at 37°C in a humidified incubator with 5% CO_2_ for the indicated time points. Following treatment, cells were lysed directly in the wells using lysis buffer (FR203‐01, Transgen), in accordance with the manufacturer's guidelines. The resulting lysates were collected, and luciferase activity was measured using a TD20/20 Luminometer (Turner Designs).

### 4.8 Total Protein Extraction and Western Blot Analysis

Cells were lysed using RIPA Lysis Buffer (Strong) (HY‐K1001, MedChemExpress), supplemented with a protease inhibitor cocktail (EDTA‐Free Protease Inhibitor Cocktail) and phosphatase inhibitor cocktails (Phosphatase Inhibitor Cocktail I and III, both 100 × in DMSO). Clarified cell extracts (10 to 30 µg) were resolved on SDS‐PAGE gels and subsequently transferred to nitrocellulose membranes (FFN08, Beyotime). Following blocking, membranes were incubated with primary antibodies specific to the target proteins. The bound secondary antibodies were detected using the enhanced chemiluminescence (ECL) method (07.10009‐50, EallBio).

### 4.9 Cell Cytotoxicity Assay

Single‐cell suspensions were prepared from freshly isolated tumor tissues by enzymatic digestion followed by filtration through a 70‐µm cell strainer. Cells were washed with staining buffer and incubated with Fc receptor blocking reagent prior to surface antibody staining. After staining, cells were washed, resuspended in staining buffer, and analyzed on an LSRFortessa flow cytometer (BD Biosciences). Flow cytometry data were analyzed using FlowJo v11 (BD Biosciences). The gating strategy was as follows. Debris was excluded based on FSC‐A vs. SSC‐A, followed by doublet discrimination using FSC‐A vs. FSC‐H to obtain singlet cells. CD45^+^ leukocytes were then gated for downstream immune‐cell analysis. For lymphoid cell analysis, CD3^+^ T cells were identified within the CD45^+^ population. CD8^+^ T cells were subsequently defined as CD3^+^CD8^+^ cells, whereas NK cells were identified as CD3^−^NK1.1^+^ cells within the CD45^+^ population. For myeloid cell analysis, CD11b^+^ myeloid cells were first gated from CD45^+^ leukocytes.

### 4.10 Pharmacological Inhibition of Proteins

To investigate the role of specific signaling pathways, cells were treated with a range of pharmacological inhibitors upon reaching 70%–80% confluence. Each inhibitor was dissolved in anhydrous DMSO and diluted to the appropriate working concentration. The following inhibitors were used: C29 (10 µm, S6597, Selleck) for TLR2 inhibition, Procyanidin B1 (30 µm, HY‐N0795, MedChemExpress) for TLR4 inhibition, MyD88‐IN‐1 (30 µm, HY‐149992, MedChemExpress) for MyD88 inhibition, Pepinh‐TRIF TFA (30 µm, HY‐P2565, MedChemExpress) for TRIF inhibition, GSK8612 (5 µm, T5540, TargetMol) for TBK1/IKKε inhibition, MNS (10 µm, HY‐78263, MedChemExpress) for SYK inhibition, and Fludarabine (10 µm, HY‐B0069, MedChemExpress) for STAT1 inhibition. Following treatment, cells were incubated at 37°C for 3 h. Control groups were treated with DMSO at a final concentration of less than 0.1%. This treatment scheme enabled the assessment of the contribution of each specific pathway to the cellular responses observed.

### 4.11 Cloning and Construction of Expression Plasmids

Mouse *Tlr4*, *Cd14*, and *Md2* cDNAs were cloned into the pcDNA3.1‐HA expression vector using a seamless cloning and assembly kit (Transgen, CU101‐01). All expression constructs were generated using standard molecular biology techniques, and the coding sequences were fully verified by sequencing. Site‐directed mutants were created using standard cloning procedures, with each mutant confirmed through sequencing. The Ifnb luciferase reporter and NF‐κB luciferase reporter plasmids were generously provided by Professor Zhengfan Jiang from Peking University. The pCMV‐VSVG and psPAX2 plasmids were obtained from Addgene.

### 4.12 Gene Silencing of Stat1 and Syk Using Short Hairpin RNA (shRNA)

Gene silencing of *Stat1* and *Syk* was achieved using the pLKO.1 plasmid vector, which contains EcoRI and AgeI restriction enzyme cutting sites. The shRNA sequences designed to knock down *Stat1* and *Syk* were sourced from the Sigma–Aldrich online database (https://www.sigmaaldrich.cn/CN/zh/product/sigma/shrna), converted by the online tools MOI (http://www.fynn‐guo.cn/seq_tool.php)^49^and are listed in Table S2. Initially, three pairs of shRNAs targeting *Stat1* and *Syk* were designed, and following validation, the pairs with the highest knockdown efficiency were selected for further experiments.

The final shRNA sequences for *Stat1*‐shRNA‐pair2 were:

- **Sense strand**: CCGGCCCTGAAGTATCTGTATCCAACTCGAGTTGGATACAGATACTTCAGGGTTTTTG
- **Antisense strand**: AATTCAAAAACCCTGAAGTATCTGTATCCAACTCGAGTTGGATACAGATACTTCAGGG

The final shRNA sequences for *Syk*‐shRNA‐pair2 were:

- **Sense strand**: CCGGGCAGGCCATCATCAGTCAGAACTCGAGTTCTGACTGATGATGGCCTGCTTTTTG
- **Antisense strand**: AATTCAAAAAGCAGGCCATCATCAGTCAGAACTCGAGTTCTGACTGATGATGGCCTGC

A nontargeting shRNA (scramble shRNA) from Sigma–Aldrich was used as a negative control. Transfections were performed at a final concentration of 10 nm using Lipofectamine RNAiMAX Transfection Reagent (catalog number 13778030; Thermo Fisher Scientific), following the manufacturer's instructions. After transfection, cells were split and selected with 5 µg/mL puromycin for 2 weeks. Stable clones were then isolated by limiting dilution and expanded for further analysis.

### 4.13 CRISPR‐Cas9 System for Syk Knockout

*Syk* knockout (KO) RAW264.7 cells were generated using the CRISPR‐Cas9 system. Guide RNAs (gRNAs) with high efficiency and specificity were designed using the SYNTHEGO online CRISPR design tool (https://www.synthego.com/products/crispr‐kits). The oligos were annealed and cloned into the lentiCRISPR v2 vector, which had been digested with the BsmBI enzyme (NEB). To generate lentivirus, 293T cells were transfected with the following plasmids: lentiCRISPR v2 (2400 ng), packaging plasmid psPAX2 (800 ng; Addgene 12260), envelope plasmid VSV‐G (800 ng; Addgene 8454), and PEI (1600 ng). The transfection mixture was incubated at 37°C for 72 h, after which viral supernatants were collected and used to infect RAW264.7 cells in the presence of polybrene (Beyotime Biotechnology, China). Forty‐eight hours post‐infection, cells were refreshed with fresh culture medium and selected with 10 µg/mL puromycin. Three pairs of sgRNAs targeting *Syk* were initially designed (see Table S2), and after validation, the pairs with the highest knockout efficiency were selected for further experiments. The successful knockout of *Syk* was validated by Western blotting. The primer sequences used for the knockout validation are as follows:

- **Sense strand**: CACCGUGAAGGGGUGCAGACAUGGC
- **Antisense strand**: AAACGCCATGTCTGCACCCCTTCAC

### 4.14 Immunoprecipitation (IP) Assay

Immunoprecipitation was performed using the ProteinIso Protein A/G Resin (DP501, TransGen Biotech) according to the manufacturer's instructions. Cells were lysed in NP‐40 lysis buffer (HY‐K1002, MedChemExpress), supplemented with protease inhibitors. The lysates were clarified by centrifugation at 12 000 rpm for 10 min at 4°C. Equal amounts of protein lysates were incubated with specific primary antibodies overnight at 4°C. Following incubation, ProteinIso Protein A/G Resin was added and rotated gently for 4 h at 4°C. The resin was washed three times with NP‐40 lysis buffer to remove nonspecific binding. Immune complexes were eluted by heating the resin in SDS‐PAGE loading buffer at 108°C for 5 min, and the resulting samples were analyzed by Western blotting.

### 4.15 Microscale Thermophoresis (MST) Assay

The interaction between TLR4 and Co68 was assessed using Microscale Thermophoresis (MST) at the State Key Laboratory, School of Pharmaceutical Sciences, Peking University Health Science Center. Recombinant human TLR4 protein was labeled with a fluorescent dye, and Co68 was prepared in a series of dilutions. The labeled TLR4 protein and Co68 were incubated together, and the mixture was loaded into capillaries for measurement. MST data were collected at 25°C, and binding affinity (K*d*) was calculated using MO.Affinity Analysis software. All data analysis was independently conducted by our research team.

### 4.16 Surface Plasmon Resonance (SPR) Assay

SPR experiments were conducted using the S‐Class label‐free molecular interaction analysis system, in collaboration with Polariton Life Sciences, to evaluate the interaction between TLR4 protein and small molecules (Co65, Co66, Co67, Co68, Co69, Co70, Co71, Co72, Co73, and Co74). TLR4 protein was immobilized onto a C5 sensor chip via amine coupling, using a protein stock solution prepared at 10 µg/mL in 1X PBST (pH 4.5), with an immobilization time of 600 s. Small molecules were dissolved in 100% DMSO at 10 mm, then diluted in 1X PBST containing 5% DMSO before injection. Each analyte was flowed over the immobilized TLR4 at a rate of 30 µL/min, with association and dissociation phases set to 60 and 120 s, respectively. Binding data were collected and analyzed to determine the kinetic parameters and affinities (K*d*) of the interactions.

### 4.17 Structure Prediction With AlphaFold

The binding interactions of the TLR4‐MD2 complex with the small molecule Co68 and the ion Co^2+^ were predicted using AlphaFold3 [48]. The amino acid sequences of TLR4 and MD2 were retrieved from the UniProt database and used to predict the structure of the TLR4‐MD2 complex. The predicted structure was evaluated for accuracy based on the confidence score (pLDDT) and aligned with known crystal structures for validation. For molecular docking, the TLR4‐MD2 complex structure predicted by AlphaFold was used as the receptor. The structure of Co68 was obtained from the PubChem database, and its geometry was optimized prior to docking. Co^2+^ binding sites were analyzed separately by including the ion in docking simulations. Molecular docking was performed using AutoDock Vina (V1.2.5) [49], and binding interactions were visualized and analyzed using PyMOL (V2.1) and Chimera (V1.18) [50]. Key residues involved in the binding of Co68 and Co^2+^ were identified and compared to evaluate their interaction patterns with the TLR4‐MD2 complex.

### 4.18 RNA‐Seq and Data Analysis

Total RNA was extracted using the high‐throughput RNA extraction kit (TIANGEN, A0123A01). Quality control, library preparation, and sequencing were performed by Suzhou GENEWIZ Biotechnology company (https://www.genewiz.com.cn/), following established standard protocols. Data analysis was carried out in‐house [51]. Sequencing raw data in FASTQ format underwent quality control using FastQC (v0.11.9) and Trim‐Galore (v0.6.4) software to generate clean data for downstream analysis. Trim‐Galore was executed with the following parameters: trim_galore –gzip –trim‐n –phred33 ‐j 7 –paired ${var}_1.fq.gz ${var}_2.fq.gz ‐o $wrk_dir/clean_result/. Clean reads were aligned to the mm10 reference genome using Subread (v2.0.0) with the following parameters [52]: subread‐align ‐i $idx_dir ‐r $cle_dir/${var}_1_val_1.fq.gz ‐R $cle_dir/${var}_2_val_2.fq.gz ‐o $aln_dir/${var}.bam ‐T 30 ‐t 0. The gene count matrix was generated using featureCounts (v2.0.0) with the following parameters [53]: featureCounts ‐p ‐t exon ‐g gene_id ‐a $gtf_dir ‐o $cnt_dir/count_refGene $aln_dir/*.bam ‐T 29. The count data was normalized using the Fragments Per Kilobase Million (FPKM) formula. Differential expression analysis was conducted using the DESeq2 R package (v1.38.3), with the thresholds set at |log2(FoldChange)| > 2 and *p*‐value < 0.05 to identify significant differentially expressed genes (DEGs) [54, 55].

### 4.19 GO Annotation and KEGG Pathway Enrichment Analysis

In this study, DEGs were subjected to enrichment analysis through Gene Ontology (GO) annotation and Kyoto Encyclopedia of Genes and Genomes (KEGG) pathway analysis [56, 57]. This analysis was performed using the clusterProfiler R package, specifically employing the enrichGO and enrichKEGG functions [57]. Alternatively, the online database DAVID (https://david.ncifcrf.gov/summary.jsp) (V6.8) was utilized for the same purpose [58]. Significance thresholds were established, with GO and KEGG terms having false discovery rates (FDR) less than 0.01 considered indicative of meaningful enrichments.

### 4.20 Gene Set Enrichment Analysis (GSEA)

Gene Set Enrichment Analysis (GSEA) is a computational approach used to determine whether predefined gene sets exhibit statistically significant and coordinated differences between two biological states. Unlike traditional methods that focus solely on differentially expressed genes (DEGs), GSEA incorporates all genes in the analysis, regardless of their significance levels. In this study, GSEA was conducted using the clusterProfiler package, and the function gseaplot2 from the same package was employed to visualize the enrichment results [57].

### 4.21 Protein‐Protein Interaction (PPI) Network Analysis

Protein‐Protein Interaction (PPI) network analysis is a computational approach used to investigate and map the interactions between proteins within a biological system. For this analysis, we utilized the online database STRING (https://string‐db.org/) with default parameters [59]. The input consisted of differentially expressed genes (DEGs), and the output was a network representing the interactions between the proteins encoded by these DEGs. Visualization of the resulting PPI network was carried out using Cytoscape software (v3.10.2) [60].

### 4.22 Transcription Factor Enrichment Analysis (TFEA)

Transcription Factor Enrichment Analysis (TFEA) is a computational method used to identify transcription factors (TFs) that may regulate differentially expressed genes (DEGs). For this analysis, we utilized iRegulon (http://iregulon.aertslab.org/tutorial.html), a Cytoscape plug‐in specifically designed for TFEA [61]. The input for iRegulon is a list of DEGs, and the output consists of a ranked list of predicted TFs based on enrichment scores. The results of TFEA can be directly visualized and further explored using Cytoscape's integrated visualization tools (v3.10.2).

### 4.23 ATAC‐Seq

We employed a high‐throughput sequencing methodology based on transposase‐mediated chromatin accessibility profiling (ATAC‐seq) [62]. This technique utilizes the specificity of Tn5 transposase to selectively cleave accessible regions of chromatin. The transposase, preloaded with DNA sequence adapters, was incubated with isolated cell nuclei, enabling the targeted insertion of adapters into open chromatin regions. Subsequently, indexed primers were utilized for PCR amplification to construct sequencing libraries. The resultant libraries, following sequencing, provided comprehensive insights into DNA regions associated with chromatin accessibility. This experiment was performed using the Hyperactive ATAC‐Seq Library Prep Kit (Vazyme Biotech, TD711), with sequencing services conducted by GENEWIZ Biotechnology company. (Suzhou, China).

### 4.24 Data Analysis of ATAC‐Seq

For ATAC‐seq high‐throughput data, sequenced by GENEWIZ Biotechnology, raw fastq data underwent quality control using Trim‐Galore (v0.6.4). Filtered reads were then aligned and quantified against the mouse genome mm10 using the bowtie2 aligner (v2.3.5.1) [63], generating SAM or BAM files. The bowtie2 alignment parameters were: bowtie2 –very‐sensitive ‐X 2000 ‐x $Bowtie2Index ‐1$cln_res/${sample}_1_val_1.fq.gz ‐2$cln_res/${sample}_2_val_2.fq.gz ‐p $PPN | samtools view ‐buSh ‐@ $PPN | samtools sort ‐@ $PPN ‐O BAM ‐o $aln_res/${sample}.sorted.bam. Sorting and indexing of SAM/BAM files were completed using samtools (v1.10) with the command [64]: samtools index ‐@ $PPN $aln_res/${sample}.sorted.bam. Peak calling was performed using MACS3 (v3.0.0a5) with default parameters [65], yielding peak files in BED format for each sample. For downstream analysis, different strategies were applied to ATAC‐seq data. ATAC‐seq peaks were merged across all samples using the bedtools merge (v2.31.1) [66] function, followed by normalization with bamCoverage (v3.3.2) in the deepTools suite [67] using the command: bamCoverage –bam $var ‐o ${var%.*}.bw –binSize 100 –normalizeUsing RPKM –effectiveGenomeSize 2864785220 –ignoreForNormalization chrM –extendReads. Differential analysis for both ATAC‐seq was conducted using csaw R package (v1.38.0) [68], while peak annotation was performed using the ChIPseeker R package (v1.34.1) [69]. Finally, peak visualization was achieved using Integrative Genomics Viewer (IGV) (v2.17.4) [70].

### 4.25 Generation and Processing of Single‐Cell RNA Sequencing (scRNA‐Seq) Data

Tumor tissues were harvested from mice following subcutaneous implantation and processed into single‐cell suspensions. Single‐cell suspensions, library construction, and sequencing were performed by Genewiz, Azenta Life Sciences, adhering to the 10 × Genomics Chromium Next GEM Single Cell 3′ Reagent Kits v3.1 protocol. Sequencing was conducted on the Illumina NovaSeq 6000 platform in paired‐end 150 bp (PE150) mode. Genewiz provided the binary FASTQ files for subsequent analysis. Raw FASTQ files were processed using the Cell Ranger pipeline (v3.1.0) for barcode demultiplexing, alignment, and unique molecular identifier (UMI) counting, generating feature‐barcode matrices. Further analysis was performed in R using Seurat (v4.3.0) [71]. The data were normalized, reduced in dimensionality, and clustered for cellular classification. Cell type annotation was performed using SingleR (v1.0) as a reference‐based annotation tool, with cluster identities further cross‐validated against canonical lineage markers curated from the CellMarker database and relevant literature [72]. Cell‐cell communication was analyzed using the CellChat package (v1.1.3) [73]. Gene set enrichment analysis (GSEA) and gene set variation analysis (GSVA) were conducted using the clusterProfiler package (v4.6.2) [57]. Pseudotime trajectory analysis was performed with Monocle3 (v1.0.0) [74]. Marker gene expression patterns were visualized using dot plots, violin plots, and heatmaps.

### 4.26 Preprocessing and Encoding of Drug‐Gene Expression Profiles Data

This study utilized a comprehensive dataset of gene expression profiles for small molecules, sourced from the Library of Integrated Network‐Based Cellular Signatures (LINCS) project (https://clue.io/lincs) [75], DrugBank [76], and ChEMBL [77], including chemical structure and bioactivity data [55]. Strict data cleaning criteria were applied: molecules with fewer than seven replicates were excluded, and molecular SMILES were parsed using RDKit (v2024.03.5). The gene expression profiles for each molecule were averaged, disregarding variations in plate, dose, treatment time, and cell line. The focus was on landmark genes associated with NF‐κB and IRF3, which are induced by lipopolysaccharide (LPS). This resulted in a final dataset comprising 7,861 valid molecules, which was split into training (5502) and test (2359) sets. The chemical structures of small molecules were represented as SMILES strings, which were converted into grammar trees and then encoded as one‐hot arrays. Dimensionality reduction was performed using a variational autoencoder (VAE), producing the input matrix (X). Simultaneously, the gene expression effects of each molecule were encoded into one‐hot arrays, with ‘1’ indicating gene upregulation and ‘0’ indicating downregulation, creating the output matrix (Y).

### 4.27 DLINP Model Architecture and Training Strategy

Small molecule structures were represented using the Simplified Molecular Input Line Entry System (SMILES). We utilized an atom‐level tokenizer to convert SMILES strings into discrete token sequences. To compensate for the lack of inherent positional information in the Transformer architecture, positional encodings (*PE*) were injected as follows:

$$P\;E_{\left\{\left(pos,2i\right)\right\}}=\sin\left(\frac{pos}{10000^{\frac{2i}{d_{\mathrm{model}}}}}\right)$$

$$\begin{array}{c} P\;E_{\left\{(pos,2i+1)\right\}}=\cos\left(\frac{pos}{10000^{\frac{2i}{d_{\mathrm{model}}}}}\right) \end{array}$$

where *pos* denotes the token position, *i* represents the dimension index, and *d_model_* is the embedding dimension (set to 512).

The backbone of the model consists of 12 stacked Transformer Encoder layers. Each layer is composed of two primary sub‐layers:

1. Multi‐Head Attention (MHA): Eight parallel attention heads were employed to capture long‐range interactions between atoms by calculating attention scores as:
$$\begin{array}{ccc} & & Attention\;\left(Q,K,V\right)=\mathrm{softmax}\left(\frac{QK^{T}}{\sqrt{\left\{d_{k}\right\}}}\right)V \end{array}$$
2. Feed‐Forward Network (FFN): This sub‐layer consists of two linear transformations with a Gaussian Error Linear Unit (GELU) activation function in between to enhance non‐linear fitting capabilities.

Each sub‐layer is complemented by Residual Connections and Layer Normalization (LN) to mitigate the vanishing gradient problem in deep networks. To derive a fixed‐length vector representation for each molecule, a Masked Global Mean Pooling layer was applied to the encoder output. This layer effectively ignores padding tokens and averages features only across valid atomic positions. The final regression module comprises two linear fully connected layers, interspersed with a Batch Normalization layer to accelerate convergence and a Dropout layer (rate = 0.2) to prevent overfitting. The model was optimized using the Mean Squared Error (MSE) loss function and the Adam optimizer, then fine‐tuned on the L1000 pre‐training dataset.

### 4.28 Evaluation of the DLINP Model Performance

The performance of the DLINP model was evaluated using several key metrics, including the confusion matrix, precision‐recall (PR) curve, receiver operating characteristic (ROC) curve, and the area under the ROC curve (AUC). These metrics provided a comprehensive assessment of the model's ability to predict gene expression profiles induced by small molecules, revealing its precision, recall, overall classification accuracy, and discriminatory power. The confusion matrix showed the distribution of true positives, true negatives, false positives, and false negatives, providing detailed information on the model's performance. The PR and ROC curves, along with the AUC, illustrated trade‐offs between sensitivity and specificity across different thresholds, further aiding evaluation of the model's predictive capabilities.

### 4.29 Statistical Analysis

All statistical analyses in this study were conducted using the Python package SciPy (https://pypi.org/project/scipy/). Results are presented as the mean ± standard error of the mean (SEM). The *p* values < 0.05 were considered statistically significant (*), *p* values < 0.01, and *p* values < 0.001 were regarded as highly statistically significant (** and ***).

## Author Contributions

Conceptualization, X.G. methodology, X.G. software, X.G. validation, X.G. and Y.Z. and X.R. and X.C. formal analysis, X.G. and Q.L. and F.Y. investigation, X.G. and Q.L. and F.Y. resources, F.Y., P.Z., Q.L. and X.R. and T.L. and X.W. data curation, X.G, F.Y and P.Z. writing – original draft, X.G and P.Z. writing – review & editing, X.G., P.Z., Q.L. and F.Y. visualization, X.G. and F.Y. supervision, X.G, P.Z., Q.L, and F.Y. project administration, X.G, P.Z., Y.X. and F.Y. funding acquisition, X.G., F.Y. and P.Z.

## Funding

This work was supported by the National Natural Science Foundation of China (82350710801, 31570891, 31872736, 32022028, 81991505, and 82201928), the China Postdoctoral Science Foundation under Grant Number 2026M791529, and the Funds for Yuebei People's Hospital—Institute of Systems Biomedicine, Peking University Health Science Center Joint Laboratory of Molecular Diagnostics, China (Grant No.: KEJIAOSHEN (2021) 17 to Prof. Pingsen Zhao and Prof. Fuping You), Natural Science Foundation of Guangdong Province, China (Grant No.: 2025A1515010579 to Prof. Pingsen Zhao), Shaoguan Municipal Science and Technology Program, China (Grant No.: 211102114530659 to Prof. Pingsen Zhao).

## Ethics Statement

The authors declare no competing interest.

## Consent

Not Applicable. This Study Did Not Involve Human Participants or Patient‐identifiable Information. All human Cell Lines and Humanized Mice Used in this Study Were Obtained From Commercial Sources

## Conflicts of Interest

The authors declare no conflicts of interest.

## Supporting information




**Supporting File 1**: advs77269‐sup‐0001‐SuppMat.zip.

**Supporting File 2**: advs77269‐sup‐0002‐SuppMat.zip.

## Data Availability

The RNA‐seq, ATAC‐seq, and scRNA‐seq datasets generated in this study are available in the Gene Expression Omnibus (GEO) database under the following accession numbers: GSE288554, GSE288553, and GSE288555, respectively.

## References

[1] D. P. Ryan , T. S. Hong , and N. Bardeesy , “Pancreatic Adenocarcinoma,” New England Journal of Medicine 371 (2014): 1039–1049. 25207767 10.1056/NEJMra1404198

[2] M. Hidalgo , “Pancreatic Cancer,” New England Journal of Medicine 362 (2010): 1605–1617. 20427809 10.1056/NEJMra0901557

[3] D. Li , K. Xie , R. Wolff , and J. L. Abbruzzese , “Pancreatic Cancer,” Lancet 363, no. 9414 (2004): 1049–1057, 10.1016/S0140-6736(04)15841-8. 15051286

[4] L. D. Wood , M. I. Canto , E. M. Jaffee , and D. M. Simeone , “Pancreatic Cancer: Pathogenesis, Screening, Diagnosis, and Treatment,” Gastroenterology 163 (2022): 386–402, 10.1053/j.gastro.2022.03.056. 35398344 PMC9516440

[5] N. Caronni , F. La Terza , F. M. Vittoria , et al., “IL‐1β+ Macrophages Fuel Pathogenic Inflammation in Pancreatic Cancer,” Nature 623, no. 7986 (2023): 415–422, 10.1038/s41586-023-06685-2. 37914939

[6] C. Garlanda and A. Mantovani , “Interleukin‐1 in Tumor Progression, Therapy, and Prevention,” Cancer Cell 39, no. 8 (2021): 1023–1027, 10.1016/j.ccell.2021.04.011. 33989512

[7] S. Das , B. Shapiro , E. A. Vucic , S. Vogt , and D. Bar‐Sagi , “Tumor Cell–Derived IL1β Promotes Desmoplasia and Immune Suppression in Pancreatic Cancer,” Cancer Research 80, no. 5 (2020): 1088–1101, 10.1158/0008-5472.CAN-19-2080. 31915130 PMC7302116

[8] Z. Zasłona , E. M. Pålsson‐McDermott , D. Menon , et al., “The Induction of Pro–IL‐1β by Lipopolysaccharide Requires Endogenous Prostaglandin E_2_ Production,” The Journal of Immunology 198 (2017): 3558–3564. 28298525 10.4049/jimmunol.1602072

[9] D. Thumkeo , S. Punyawatthananukool , S. Prasongtanakij , et al., “PGE_2_‐EP2/EP4 Signaling Elicits Immunosuppression by Driving the mregDC‐Treg Axis in Inflammatory Tumor Microenvironment,” Cell Reports 39, no. 10 (2022): 110914, 10.1016/j.celrep.2022.110914. 35675777

[10] S. B. Lacher , J. Dörr , G. P. de Almeida , et al., “PGE_2_ Limits Effector Expansion of Tumour‐Infiltrating Stem‐Like CD8^+^ T Cells,” Nature 629, no. 8011 (2024): 417–425, 10.1038/s41586-024-07254-x. 38658748 PMC11078747

[11] M. Morotti , A. J. Grimm , H. C. Hope , et al., “PGE_2_ Inhibits TIL Expansion by Disrupting IL‐2 Signalling and Mitochondrial Function,” Nature 629, no. 8011 (2024): 426–434, 10.1038/s41586-024-07352-w. 38658764 PMC11078736

[12] J. Bhattacharyya , J. J. Bellucci , I. Weitzhandler , et al., “A Paclitaxel‐Loaded Recombinant Polypeptide Nanoparticle Outperforms Abraxane in Multiple Murine Cancer Models,” Nature Communications 6, no. 1 (2015): 7939, 10.1038/ncomms8939. PMC4753781 26239362

[13] L. Volk‐Draper , K. Hall , C. Griggs , et al., “Paclitaxel Therapy Promotes Breast Cancer Metastasis in a TLR4‐Dependent Manner,” Cancer Research 74, no. 19 (2014): 5421–5434, 10.1158/0008-5472.CAN-14-0067. 25274031 PMC4185415

[14] L. Zhu and L. Chen , “Progress in Research on Paclitaxel and Tumor Immunotherapy,” Cellular & Molecular Biology Letters 24, no. 1 (2019): 40, 10.1186/s11658-019-0164-y. 31223315 PMC6567594

[15] S. Rajput , L. D. Volk‐Draper , and S. Ran , “TLR4 is a Novel Determinant of the Response to Paclitaxel in Breast Cancer,” Molecular Cancer Therapeutics 12, no. 8 (2013): 1676–1687, 10.1158/1535-7163.MCT-12-1019. 23720768 PMC3742631

[16] C. W. Wanderley , D. F. Colón , J. P. M. Luiz , et al., “Paclitaxel Reduces Tumor Growth by Reprogramming Tumor‐Associated Macrophages to an M1 Profile in a TLR4‐Dependent Manner,” Cancer Research 78, no. 20 (2018): 5891–5900, 10.1158/0008-5472.CAN-17-3480. 30104241

[17] D. J. Perkins , K. Richard , A.‐M. Hansen , et al., “Autocrine–Paracrine Prostaglandin E2 Signaling Restricts TLR4 Internalization and TRIF Signaling,” Nature Immunology 19, no. 12 (2018): 1309–1318, 10.1038/s41590-018-0243-7. 30397349 PMC6240378

[18] A. Piaszyk‐Borychowska , L. Széles , A. Csermely , et al., “Signal Integration of IFN‐I and IFN‐II With TLR4 Involves Sequential Recruitment of STAT1‐Complexes and NFκB to Enhance Pro‐Inflammatory Transcription,” Frontiers in Immunology 10 (2019): 01253, 10.3389/fimmu.2019.01253. PMC6558219 31231385

[19] S. I. Grivennikov , F. R. Greten , and M. I. Karin , “Immunity, Inflammation, and Cancer,” Cell 140 (2010): 883–899, 10.1016/j.cell.2010.01.025. 20303878 PMC2866629

[20] T. Lawrence , “The Nuclear Factor NF‐ B Pathway in Inflammation,” Cold Spring Harbor Perspectives in Biology 1, no. 6 (2009): a001651–a001651, 10.1101/cshperspect.a001651. 20457564 PMC2882124

[21] R. Burbidge , M. Trotter , B. Buxton , and S. Holden , “Drug Design by Machine Learning: Support Vector Machines for Pharmaceutical Data Analysis,” Computers & Chemistry 26, no. 1 (2001): 5–14, 10.1016/S0097-8485(01)00094-8. 11765851

[22] Y. Tang , J. Y. Kim , C. K. M. IP , et al., “Data‐Driven Discovery of Innate Immunomodulators via Machine Learning‐Guided High Throughput Screening,” Chemical Science 14, no. 44 (2023): 12747–12766, 10.1039/D3SC03613H. 38020385 PMC10646978

[23] Z. Tanoli , M. Vähä‐Koskela , and T. Aittokallio , “Artificial Intelligence, Machine Learning, and Drug Repurposing in Cancer,” Expert Opinion on Drug Discovery 16, no. 9 (2021): 977–989, 10.1080/17460441.2021.1883585. 33543671

[24] S. Vatansever , A. Schlessinger , D. Wacker , et al., “Artificial Intelligence and Machine Learning‐Aided Drug Discovery in Central Nervous System Diseases: State‐Of‐The‐Arts and Future Directions,” Medicinal Research Reviews 41, no. 3 (2021): 1427–1473, 10.1002/med.21764. 33295676 PMC8043990

[25] M. Koromina , M. T. Pandi , and G. P. Patrinos , “Rethinking Drug Repositioning and Development With Artificial Intelligence, Machine Learning, and Omics,” Omics: A journal of Integrative Biology 23 (2019): 539–548. 31651216 10.1089/omi.2019.0151

[26] N. T. Issa , V. Stathias , S. Schürer , and S. Dakshanamurthy , “Machine and Deep Learning Approaches for Cancer Drug Repurposing,” Seminars in Cancer Biology 68 (2021): 132–142, 10.1016/j.semcancer.2019.12.011. 31904426 PMC7723306

[27] A. Mayr , G. Klambauer , T. Unterthiner , et al., “Large‐Scale Comparison of Machine Learning Methods for Drug Target Prediction on ChEMBL,” Chemical Science 9, no. 24 (2018): 5441–5451, 10.1039/C8SC00148K. 30155234 PMC6011237

[28] P. Dragan , M. Merski , S. Wiśniewski , S. G. Sanmukh , and D. Latek , “Chemokine Receptors—Structure‐Based Virtual Screening Assisted by Machine Learning,” Pharmaceutics 15 (2023): 516. 36839838 10.3390/pharmaceutics15020516 PMC9965785

[29] B. Hie , B. D. Bryson , and B. Berger , “Leveraging Uncertainty in Machine Learning Accelerates Biological Discovery and Design,” Cell systems 11 (2020): 461–477. 33065027 10.1016/j.cels.2020.09.007

[30] J. Vamathevan , D. Clark , P. Czodrowski , et al., “Applications of Machine Learning in Drug Discovery and Development,” Nature Reviews Drug Discovery 18, no. 6 (2019): 463–477, 10.1038/s41573-019-0024-5. 30976107 PMC6552674

[31] K. T. Butler , D. W. Davies , H. Cartwright , O. Isayev , and A. Walsh , “Machine Learning for Molecular and Materials Science,” Nature 559, no. 7715 (2018): 547–555, 10.1038/s41586-018-0337-2. 30046072

[32] S. Yang , P. Varghese , E. Stephenson , K. Tu , and J. Gronsbell , “Machine Learning Approaches for Electronic Health Records Phenotyping: A Methodical Review,” Journal of the American Medical Informatics Association 30, no. 2 (2023): 367–381, 10.1093/jamia/ocac216. 36413056 PMC9846699

[33] A. Elewaut , G. Estivill , F. Bayerl , et al., “Cancer Cells Impair Monocyte‐Mediated T Cell Stimulation to Evade Immunity,” Nature 637 (2024): 716–725, 10.1038/s41586-024-08257-4. 39604727 PMC7617236

[34] S. Cheng , Z. Li , R. Gao , et al., “A Pan‐Cancer Single‐Cell Transcriptional Atlas of Tumor Infiltrating Myeloid Cells,” Cell 184, no. 3 (2021): 792–809.e23, 10.1016/j.cell.2021.01.010. 33545035

[35] R. Bill , P. Wirapati , M. Messemaker , et al., “CXCL9:SPP1 Macrophage Polarity Identifies a Network of Cellular Programs That Control Human Cancers,” Science 381 (2023): 515–524. 37535729 10.1126/science.ade2292 PMC10755760

[36] O. Takeuchi and S. Akira , “Pattern Recognition Receptors and Inflammation,” Cell 140, no. 6 (2010): 805–820, 10.1016/j.cell.2010.01.022. 20303872

[37] Q. Guo , Y. Zhao , J. Li , et al., “Induction of Alarmin S100A8/A9 Mediates Activation of Aberrant Neutrophils in the Pathogenesis of COVID‐19,” Cell Host & Microbe 29, no. 2 (2021): 222–235.e4, 10.1016/j.chom.2020.12.016. 33388094 PMC7762710

[38] Y. Zhao , M. Kuang , J. Li , et al., “SARS‐CoV‐2 Spike Protein Interacts With and Activates TLR41,” Cell Research 31, no. 7 (2021): 818–820, 10.1038/s41422-021-00495-9. 33742149 PMC7975240

[39] A. Mantovani , P. Allavena , A. Sica , and F. Balkwill , “Cancer‐Related Inflammation,” Nature 454, no. 7203 (2008): 436–444, 10.1038/nature07205. 18650914

[40] U. Ohto , K. Fukase , K. Miyake , and T. Shimizu , “Structural Basis of Species‐Specific Endotoxin Sensing by Innate Immune Receptor TLR4/MD‐2,” Proceedings of the National Academy of Sciences 109, no. 19 (2012): 7421–7426, 10.1073/pnas.1201193109. PMC3358893 22532668

[41] B. S. Park , D. H. Song , H. M. Kim , B.‐S. Choi , H. Lee , and J.‐O. Lee , “The Structural Basis of Lipopolysaccharide Recognition by the TLR4–MD‐2 Complex,” Nature 458, no. 7242 (2009): 1191–1195, 10.1038/nature07830. 19252480

[42] V. Mata‐Haro , C. Cekic , M. Martin , P. M. Chilton , C. R. Casella , and T. C. Mitchell , “The Vaccine Adjuvant Monophosphoryl Lipid A as a TRIF‐Biased Agonist of TLR4,” Science 316, no. 5831 (2007): 1628–1632, 10.1126/science.1138963. 17569868

[43] A. Ciesielska , M. Matyjek , and K. Kwiatkowska , “TLR4 and CD14 Trafficking and its Influence on LPS‐Induced Pro‐Inflammatory Signaling,” Cellular and Molecular Life Sciences 78, no. 4 (2021): 1233–1261, 10.1007/s00018-020-03656-y. 33057840 PMC7904555

[44] S. Liu , Y. Liao , B. Chen , et al., “Critical Role of Syk‐Dependent STAT1 Activation in Innate Antiviral Immunity,” Cell Reports 34 (2021): 108627. 33472080 10.1016/j.celrep.2020.108627

[45] Y.‐C. Lin , D.‐Y. Huang , C.‐L. Chu , Y.‐L. Lin , and W.‐W. Lin , “The Tyrosine Kinase Syk Differentially Regulates Toll‐Like Receptor Signaling Downstream of the Adaptor Molecules TRAF6 and TRAF3,” Science Signaling 6 (2013): 2003973. 10.1126/scisignal.2003973 23962979

[46] S. Joshi , “New Insights Into SYK Targeting in Solid Tumors,” Trends in Pharmacological Sciences 45, no. 10 (2024): 904–918, 10.1016/j.tips.2024.08.006. 39322438 PMC11984332

[47] J. J. Balic , H. Albargy , K. Luu , et al., “STAT3 Serine Phosphorylation is Required for TLR4 Metabolic Reprogramming and IL‐1β Expression,” Nature Communications 11, no. 1 (2020): 3816, 10.1038/s41467-020-17669-5. PMC7393113 32732870

[48] J. Jumper , R. Evans , A. Pritzel , et al., “Highly Accurate Protein Structure Prediction With AlphaFold,” Nature 596, no. 7873 (2021): 583–589, 10.1038/s41586-021-03819-2. 34265844 PMC8371605

[49] O. Trott and A. J. Olson , “AutoDock Vina: Improving the Speed and Accuracy of Docking With a New Scoring Function, Efficient Optimization, and Multithreading,” Journal of Computational Chemistry 31, no. 2 (2010): 455–461, 10.1002/jcc.21334. 19499576 PMC3041641

[50] E. F. Pettersen , T. D. Goddard , C. C. Huang , et al., “UCSF Chimera—A Visualization System for Exploratory Research and Analysis,” Journal of Computational Chemistry 25, no. 13 (2004): 1605–1612, 10.1002/jcc.20084. 15264254

[51] X. Guo , Y. Zhao , and F. You , “MOI is a Comprehensive Database Collecting Processed Multi‐Omics Data Associated With Viral Infection,” Scientific Reports 14 (2024): 14725. 38926513 10.1038/s41598-024-65629-6 PMC11208532

[52] Y. Liao , G. K. Smyth , and W. Shi , “The Subread Aligner: Fast, Accurate and Scalable Read Mapping by Seed‐and‐Vote,” Nucleic Acids Research 41, no. 10 (2013): e108–e108, 10.1093/nar/gkt214. 23558742 PMC3664803

[53] Y. Liao , G. K. Smyth , and W. Shi , “FeatureCounts: An Efficient General Purpose Program for Assigning Sequence Reads to Genomic Features,” Bioinformatics 30, no. 7 (2014): 923–930, 10.1093/bioinformatics/btt656. 24227677

[54] X. Guo , Y. Zhao , and F. You , “Identification and Characterization of Endogenous Retroviruses Upon SARS‐CoV‐2 Infection,” Frontiers in Immunology 15 (2024): 1294020. 38646531 10.3389/fimmu.2024.1294020 PMC11026653

[55] X. Guo , Y. Zhao , X. Rong , et al., “VDLIN: A Deep Learning‐Based Platform for Methylcobalamin‐Inspired Immunomodulatory Compound Screening,” Advanced Science 13 (2025): 13775, 10.1002/advs.202413775. PMC12866821 41144841

[56] M. Kanehisa and S. Goto , “KEGG: Kyoto Encyclopedia of Genes and Genomes,” Nucleic Acids Research 28, no. 1 (2000): 27–30, http://www.genome.ad.jp/kegg/. 10592173 10.1093/nar/28.1.27 PMC102409

[57] G. Yu , L. G. Wang , Y. Han , and Q. Y. He , “ClusterProfiler: An R Package for Comparing Biological Themes Among Gene Clusters,” OMICS: A Journal of Integrative Biology 16, no. 5 (2012): 284–287, 10.1089/omi.2011.0118. 22455463 PMC3339379

[58] G. Dennis , B. T. Sherman , D. A. Hosack , et al., “DAVID: Database for Annotation, Visualization, and Integrated Discovery,” Genome Biology 4, no. 9 (2003): R60, http://dot.ped.med.umich.edu:2000/.12734009

[59] D. Szklarczyk , A. L. Gable , D. Lyon , et al., “STRING v11: Protein–Protein Association Networks With Increased Coverage, Supporting Functional Discovery in Genome‐Wide Experimental Datasets,” Nucleic Acids Research 47, no. D1 (2019): D607–D613, 10.1093/nar/gky1131. 30476243 PMC6323986

[60] P. Shannon , A. Markiel , O. Ozier , et al., “Cytoscape: A Software Environment for Integrated Models of Biomolecular Interaction Networks,” Genome Research 13, no. 11 (2003): 2498–2504, 10.1101/gr.1239303. 14597658 PMC403769

[61] R. Janky , A. Verfaillie , H. Imrichová , et al., “iRegulon: From a Gene List to a Gene Regulatory Network Using Large Motif and Track Collections,” PLoS Computational Biology 10, no. 7 (2014): 1003731, 10.1371/journal.pcbi.1003731. PMC4109854 25058159

[62] J. D. Buenrostro , B. Wu , H. Y. Chang , and W. J. Greenleaf , “ATAC‐Seq: A Method for Assaying Chromatin Accessibility Genome‐Wide,” Current Protocols in Molecular Biology 109 (2015): 21–29. 10.1002/0471142727.mb2129s109 PMC4374986 25559105

[63] B. Langmead , C. Trapnell , M. Pop , and S. L. Salzberg , “Ultrafast and Memory‐Efficient Alignment of Short DNA Sequences to the Human Genome,” Genome Biology 10, no. 3 (2009): R25, 10.1186/gb-2009-10-3-r25. 19261174 PMC2690996

[64] H. Li , B. Handsaker , A. Wysoker , et al., “The Sequence Alignment/Map Format and SAMtools,” Bioinformatics 25, no. 16 (2009): 2078–2079, 10.1093/bioinformatics/btp352. 19505943 PMC2723002

[65] Y. Zhang , T. Liu , C. A. Meyer , et al., “Model‐Based Analysis of ChIP‐Seq (MACS),” Genome Biology 9, no. 9 (2008): R137, 10.1186/gb-2008-9-9-r137. 18798982 PMC2592715

[66] A. R. Quinlan and I. M. Hall , “BEDTools: A Flexible Suite of Utilities for Comparing Genomic Features,” Bioinformatics 26, no. 6 (2010): 841–842, 10.1093/bioinformatics/btq033. 20110278 PMC2832824

[67] F. Ramírez , F. Dündar , S. Diehl , B. A. Grüning , and T. Manke , “DeepTools: A Flexible Platform for Exploring Deep‐Sequencing Data,” Nucleic Acids Research 42 (2014): W187–W191. 24799436 10.1093/nar/gku365 PMC4086134

[68] A. T. L. Lun and G. K. Smyth , “Csaw: A Bioconductor Package for Differential Binding Analysis of ChIP‐Seq Data Using Sliding Windows,” Nucleic Acids Research 44, no. 5 (2015): 45, 10.1093/nar/gkv1191. 26578583 PMC4797262

[69] G. Yu , L. G. Wang , and Q. Y. He , “ChIP Seeker: An R/Bioconductor Package for ChIP Peak Annotation, Comparison and Visualization,” Bioinformatics 31, no. 14 (2015): 2382–2383, 10.1093/bioinformatics/btv145. 25765347

[70] J. T. Robinson , H. Thorvaldsdóttir , W. Winckler , et al., “Integrative Genomics Viewer,” Nature Biotechnology 29, no. 1 (2011): 24–26, 10.1038/nbt.1754. PMC3346182 21221095

[71] R. Satija , J. A. Farrell , D. Gennert , A. F. Schier , and A. Regev , “Spatial Reconstruction of Single‐Cell Gene Expression Data,” Nature Biotechnology 33, no. 5 (2015): 495–502, 10.1038/nbt.3192. PMC4430369 25867923

[72] D. Aran , A. P. Looney , L. Liu , et al., “Reference‐Based Analysis of Lung Single‐Cell Sequencing Reveals a Transitional Profibrotic Macrophage,” Nature Immunology 20, no. 2 (2019): 163–172, 10.1038/s41590-018-0276-y. 30643263 PMC6340744

[73] S. Jin , C. F. Guerrero‐Juarez , L. Zhang , et al., “Inference and Analysis of Cell‐Cell Communication Using CellChat,” Nature Communications 12, no. 1 (2021): 1088, 10.1038/s41467-021-21246-9. PMC7889871 33597522

[74] C. Trapnell , D. Cacchiarelli , J. Grimsby , et al., “The Dynamics and Regulators of Cell Fate Decisions are Revealed by Pseudotemporal Ordering of Single Cells,” Nature Biotechnology 32, no. 4 (2014): 381–386, 10.1038/nbt.2859. PMC4122333 24658644

[75] A. Subramanian , R. Narayan , S. M. Corsello , et al., “A Next Generation Connectivity Map: L1000 Platform and the First 1,000,000 Profiles,” Cell 171, no. 6 (2017): 1437–1452.e17, 10.1016/j.cell.2017.10.049. 29195078 PMC5990023

[76] D. S. Wishart , “DrugBank: A Comprehensive Resource for in Silico Drug Discovery and Exploration,” Nucleic Acids Research 34, no. 90001 (2006): D668–D672, 10.1093/nar/gkj067. 16381955 PMC1347430

[77] D. Mendez , A. Gaulton , A. P. Bento , et al., “ChEMBL: Towards Direct Deposition of Bioassay Data,” Nucleic Acids Research 47, no. D1 (2019): D930–D940, 10.1093/nar/gky1075. 30398643 PMC6323927

