# Supplementary material for: Uncoupling Type I Interferon Benefits From Inflammatory Toxicity: Transformer‐Prioritized Precision Agonists for Potent and Safer Cancer Immunotherapy
Source: Adv Sci (Weinh). 2026 Aug 24:e77269. Online ahead of print. doi: 10.1002/advs.77269 (PMC13502280; doi:10.1002/advs.77269)

Myeloid representative KO

Source: 20220615_KO-M1_003.fcs; events: 11,555; representative raw scatter plots. Gate boundaries should be exported from the final FlowJo workspace.

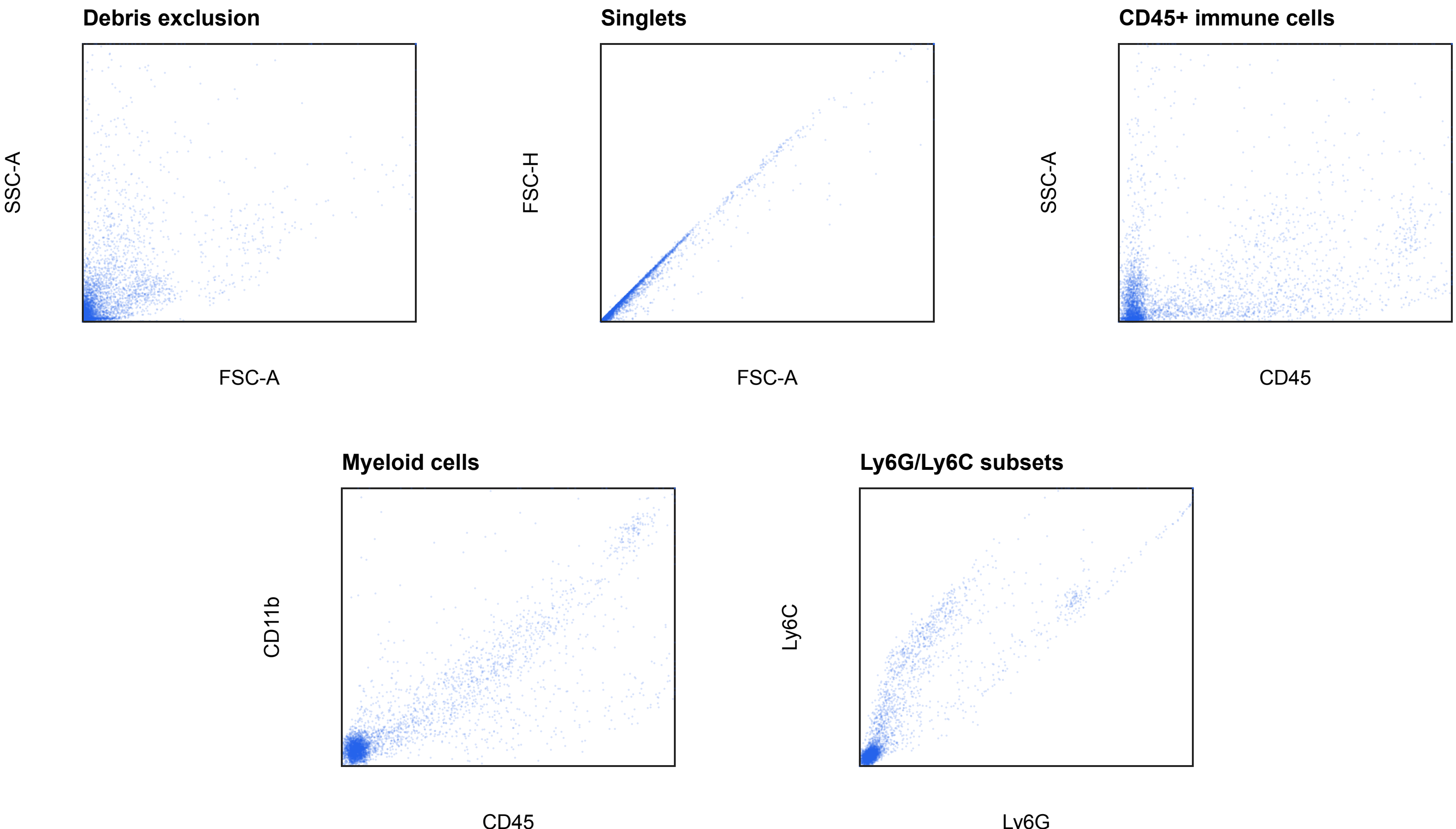

Supplement: Supplementary file 2 [file ADVS-9999-e77269-s002.zip › FACS/flow_representative_plots/Myeloid_representative_Co68.pdf]
